# Myeloid ectopic viral integration site 2 accelerates the progression of Alzheimer's disease

**DOI:** 10.1111/acel.14260

**Published:** 2024-07-12

**Authors:** Yuting Cui, Xiaomin Zhang, Jing Liu, Yuli Hou, Qiao Song, Min Cao, Jingjing Zhang, Xiaoling Wang, Congcong Liu, Peichang Wang, Yaqi Wang

**Affiliations:** ^1^ Clinical Laboratory of Xuanwu Hospital, Capital Medical University Beijing People's Republic of China; ^2^ Department of Clinical Laboratory Beijing Huairou Hospital Beijing People's Republic of China

**Keywords:** β‐Site amyloid precursor protein cleaving enzyme 1, Alzheimer's disease, myeloid ectopic viral integration site 2, transcription pathway

## Abstract

Amyloid plaques, a major pathological hallmark of Alzheimer's disease (AD), are caused by an imbalance between the amyloidogenic and non‐amyloidogenic pathways of amyloid precursor protein (APP). BACE1 cleavage of APP is the rate‐limiting step for amyloid‐β production and plaque formation in AD. Although the alteration of BACE1 expression in AD has been investigated, the underlying mechanisms remain unknown. In this study, we determined MEIS2 was notably elevated in AD models and AD patients. Alterations in the expression of MEIS2 can modulate the levels of BACE1. MEIS2 downregulation improved the learning and memory retention of AD mice and decreased the number of amyloid plaques. MEIS2 binds to the BACE1 promoter, positively regulates BACE1 expression, and accelerates APP amyloid degradation in vitro. Therefore, our findings suggest that MEIS2 might be a critical transcription factor in AD, since it regulates BACE1 expression and accelerates BACE1‐mediated APP amyloidogenic cleavage. MEIS2 is a promising early intervention target for AD treatment.

## Graphical abstract

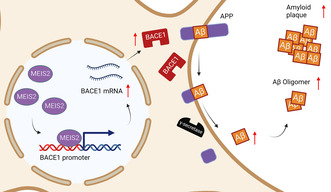

**List of abbreviations**
AAV: adeno‐associated viral vector
AD: Alzheimer's disease
ADAM10: A disintegrin and metalloproteinase 10
APP/PS1: APPSwe/PS1dE9
Aβ: β‐amyloid
BACE1: β‐Site amyloid precursor protein cleaving enzyme 1
ChIP: chromatin immunoprecipitation
DAT: dementia of the Alzheimer type
DEGs: differentially expressed genes
MCI: mild cognitive impairment
MEIS2: myeloid ectopic viral integration site 2
MWM: Morris water maze
NCSTN: nicastrin
NOR: novel object recognition
PSEN1: presenilin 1
sAPPβ: soluble beta fragment of amyloid precursor protein

## 1 INTRODUCTION

Alzheimer's disease (AD) is a chronic, progressive, neurodegenerative disorder that is the most prevalent cause of dementia. According to a report by AD International, about 150 million people suffer from AD worldwide (Alzheimer's & Dementia, 2021; Scheltens et al., 2021). AD has multiple risk factors that cause various pathological complications, including neuroinflammation, synapse loss, oxidative stress, and dysfunctional glucose metabolism (Butterfield & Halliwell, 2019; Tönnies & Trushina, 2017). Typical AD pathological changes in the brain include the accumulation of tau tangles and senile plaques composed of β‐amyloid (Aβ). Studying Aβ regulation could provide insight into developing new strategies to effectively delay or reverse AD.

Aβ is generated from the cleavage of amyloid precursor protein (APP) by β‐site amyloid precursor protein cleaving enzyme 1 (BACE1), the rate‐limiting enzyme in this pathway (Hampel et al., 2021; Zhu et al., 2019). BACE1 cleaves APP to liberate the soluble beta fragment of amyloid precursor protein (sAPPβ) and the 99‐amino acid peptide of the C‐terminal fragment of APP (CTF‐β or C99). CTF‐β is then cleaved by γ‐secretase to produce Aβ and a membrane‐binding fragment (Hampel et al., 2021; Zheng et al., 2022).

Therefore, BACE1 is a prime target for slowing Aβ production in early AD. Many studies have used BACE1 inhibitors to reverse disease progression and associated neuronal damage in AD. While almost all the inhibitors have failed in clinical trials, this does not mean that BACE1 has lost its research value (Moussa‐Pacha et al., 2020). Fundamentally, there is no specific treatment for AD as it is exact biological mechanisms have not been well elucidated (Patel et al., 2022; Vassar, 2014).

The myeloid ectopic viral integration site 2 (Meis2) gene encodes a transcription factor that belongs to the superclass of three‐amino acid loop extension (TALE) proteins (Durán Alonso et al., 2021). MEIS proteins have common structural features: two short alpha helices and a TALE with a DNA‐binding function. The C terminus of MEIS proteins has a transcriptional activation domain (Schulte & Geerts, 2019). Recent studies showed that MEIS2 regulates striatal neuron development (Su et al., 2022), the maintenance of retinal progenitor pool (Dupacova et al., 2021) and palatal osteogenesis (Wang, Tang, et al., 2020). The sequencing of our group found that MEIS2 was differentially expressed in the hippocampus of APP/PS1 mice, and predictively bind to the BACE1 promoter, suggesting that MEIS2 might play an important role in AD.

In this study, we investigated changes in MEIS2 levels in AD and examined the effects of MEIS2 on the pathological progression in APP/PS1 mice. In addition, the mechanism by which MEIS2 regulates AD progression was elucidated.

## 2 MATERIALS AND METHODS

### 2.1 Human samples

Hippocampal and temporal cortex samples from post‐mortem AD and age‐matched cases were obtained from the Chinese Brain Bank Center (CBBC). The specimen information is listed in Table S1. CSF samples were taken from 155 participants comprised three subgroups: normal cognition, mild cognitive impairment (MCI) due to AD, and dementia stage of Alzheimer's disease (DAT). The normal cognition group included 45 patients, excluding those with other types of dementia, multisystem atrophy, cerebral haemorrhage, Parkinson's disease, progressive supranuclear palsy and other neuropsychiatric diseases. Forty‐five individuals with MCI and 65 individuals with DAT were diagnosed by a neurologist at the Xuanwu Hospital in Beijing according to the NINCDS‐ADRDA criteria (Koldamova et al., 2005). Serum samples from 250 participants comprised three subgroups: 95 healthy control (HC) individuals were enrolled among individuals referring to healthy older persons from physical examinations at the Xuanwu Hospital; 60 individuals with MCI and 95 individuals with DAT were diagnosed as described above. CSF and serum samples were partially packed and frozen at −80°C.

The study design was approved by the Ethics Committee of Xuanwu Hospital of Capital Medical University and was conducted following the guidelines of the Declaration of Helsinki.

### 2.2 Animal treatment

We obtained 2‐, 5‐, and 8‐month male APP_Swe_/PS1_dE9_ (APP/PS1) transgenic mice (*n* = 6 for each group) and age‐matched C57BL/6 (wild‐type) mice (*n* = 6 for each group) from the Zhishan Institute of Health Medicine Co., Ltd (Beijing, China). Animal care and handling were performed according to the NIH animal care guidelines, the Declaration of Helsinki, and the Xuanwu Hospital guidelines.

For adeno‐associated virus vector carrying MEIS2 cDNA (AAV_oeMEIS2_) and MEIS2 shRNA (AAV_shMEIS2_) treatment, 6‐month‐old mice (*n* = 6 for each group) were anaesthetised with 1% pentobarbital sodium (0.1 mL/20 g, i.p.). They were then mounted on the stereotaxic apparatus and 3 × 10^9^ viral genomes/site were microinjected into the hippocampus (anterior–posterior: −2.0 mm, medial‐lateral: ±1.5 mm, dorsal‐ventral: −2.0 mm). After 4 weeks of recovery, the mice were subjected to behavioural examinations and sacrificed. The brains were isolated and stored at −80°C for further tests.

All animal experiments were conducted per the Regulations of Beijing Municipality on the Administration of Laboratory Animals and were approved by the Bioethics Committee of Xuanwu Hospital of Capital Medical University.

### 2.3 Cell culture and transfection

N2a and HT22 cells were purchased from American Type Culture Collection. N2a and HT22 cells were cultured in Dulbecco's modified Eagle's medium (DMEM, Biological Industries) supplemented with 10% fetal bovine serum (FBS, Biological Industries) and 1% penicillin/streptomycin (Biological Industries). HT22^APP^ cells stably transfected with the human Swedish mutant APP were cultured in a complete DMEM medium with 2 μg/mL puromycin. Cells were maintained at 37°C in a 5% CO_2_ humidified atmosphere.

For plasmid transfection, HT22 cells were seeded into 24‐well culture plates at a density of 1 × 10^5^ cells/well. When the confluency reached 70%–90%, 0.8 μg DNA, 2.0 μL Lipofectamine (Lip) 2000 (Invitrogen) and 100 μL Opti‐MEM I ReLipced Serum Medium (Gibco) were mixed and incubated for 20 min at room temperature. The DNA‐Lip2000 complex was then added to the inoculated cells, and the culture plate was gently shaken back and forth for restoration. After 4–6 h incubation, the medium was changed, and the cells were cultured for 48 h. The cells were collected for subsequent analyses.

Primary neurons were prepared from embryonic Day 14.5 (E14.5) C57BL/6 mice. The brains were detached and digested with 2 mg/mL papain (Sangon Biotech) for 10 min. After adding 10% FBS to terminate digestion, primary neurons were plated on poly D‐lysine‐coated 6‐well plates at a density of 1 × 10^5^ cells/well and cultured in DMEM for 4 h. The medium was replaced with Neurobasal™ medium (Gibco) supplemented with 2% B‐27 (Gibco) and glutamine (0.5 mM, Gibco). Half of the medium was replaced every 3 days.

### 2.4 Plasmids, lentivirus, and adeno‐associated virus

Multiple vectors were constructed to overexpress or knock down MEIS2 in vivo and in vitro. The transcript MEIS2A, which is highly expressed in adult mouse brain tissue, was used in our research (Figure S2). The mouse MEIS2 (NM_001159567) overexpression plasmid (oeMEIS2), luciferase reporter gene of the mouse *Bace1* promoter plasmid, and site‐directed mutants of the mouse *Bace1* promoter plasmids were purchased from Syngentech. The mouse short‐hairpin RNA targeting MEIS2 (shRNA‐MEIS2) lentivirus was purchased from Hanbio Biotechnology (Shanghai, China). Mouse shRNA‐BACE1 (LV_shBACE1_) and human APP_swe_ overexpression lentivirus were purchased from Syngentech. Adeno‐associated viruses expressing MEIS2 (AAV_oeMEIS2_) and MEIS2 small hairpin RNA (AAV_shMEIS2_) were constructed by Hanbio Biotechnology. The shRNA target sequences are listed in Table S4.

### 2.5 Western blot

Cultured cells and brain tissue samples were lysed in a homogeneous RIPA buffer. Protein concentrations were measured using the BCA method (Thermo Fisher Scientific). Samples were prepared by mixing with 5 × loading buffer at 95°C for 10 min. Equal amounts of total protein were separated using 8% or 10% Tris‐glycine SDS‐PAGE and transferred onto polyvinylidene difluoride membranes (EMD Millipore). The membranes were incubated with blocking buffer (5% non‐fat milk in Tris Buffer Saline Tween‐20 [TBST]) for 1 h at room temperature; primary antibodies in blocking buffer were added and incubated with the membranes overnight at 4°C. The primary antibodies used were against MEIS2 (1:1000, rabbit 11550‐1‐AP, Proteintech), BACE1 (1:1000, rabbit, 12807‐1‐AP, Proteintech), ADAM10 (1:1000, rabbit 25900‐1‐AP, Proteintech), NCSTN (1:1000, rabbit 14071‐1‐AP, Proteintech), PSEN1 (1:1000, rabbit 16163‐1‐AP, Proteintech), APP (1:1000, rabbit 25524‐1‐AP, Proteintech), and GAPDH (1:5000, mouse, 60004‐1‐Ig, Proteintech).

### 2.6 ELISA

Aβ_1‐40_ (R&D), Aβ_1‐42_ (Mlbio), and sAPPβ (Mlbio) levels in mouse brain extracts and cell culture media were determined using commercially available enzyme‐linked ELISA kits. MEIS2 levels in the serums of humans and mice were measured using a sandwich ELISA kit (Abebio).

### 2.7 Real‐time quantitative polymerase chain reaction (RT‐qPCR)

Total RNA was isolated from tissues and cells using TRIzol reagent (Invitrogen). Reverse transcription was performed using a 5 × All‐In‐One RT Master Mix (ABM) according to the manufacturer's protocol. The cDNA of MEIS2, BACE1, GAPDH, and β‐actin were detected by RT‐qPCR using TB Green™ Premix Ex Taq™ II (TaKaRa) with the LightCycler 480 system (Roche). The reaction conditions were optimised based on prior studies (Bum‐Erdene et al., 2019). After that, relative quantitative analysis of gene expression was calculated using the 2^−ΔΔCt^ method with GAPDH or β‐actin as control. The primers used are listed in Table S5.

### 2.8 Morris water maze

The Morris water maze test was conducted in a circular tank filled with warm water made opaque by adding milk, and divided into four reference quadrants of equal area. The identity shapes were pasted onto the inner edges of the pool. The hidden platform training lasted for 5 days. In this test, the hidden platform was placed in the fourth quadrant, and the animals were placed in the pool at random points. The times at which the animals found the platform and their swimming paths were recorded. In the platform test phase on the seventh day, the hidden platform was removed, and each subject was given 60 s to determine the original location of the platform. The time spent in each area and the times of crossings over the platform location were recorded.

### 2.9 Open field test

The open field test was performed in a closed flat cage to observe the activity of the subjects. The cage was divided into a central square and the surrounding area. The animals were placed in the center of the box for free exploration and recorded for 10 min in a quiet environment.

### 2.10 Novel object recognition test

The novel object recognition (NOR) test was based on previous research (Krammes et al., 2020). Two objects were placed in the back left and right corners of the home cages for 24 h for familiarisation. In the familiarisation trial, mice were placed at the midpoint of the wall, opposite the samples and facing away from the objects. After 1 h of recording, animals were allowed to rest for 24 h. Object memory was tested using the same procedure; however, one object was replaced with a novel object in its original place. When the animal's snout contacted either object, exploration was recorded and calculated using the recognition index with Formula (1). T_novel_ and T_familiar_ indicate the exploration time spent by animals with novel and familiar objects, respectively.

(1) $$\mathrm{RI}=T_{\text{novel}}/\left(T_{\text{novel}}+T_{\text{familiar}}\right)$$

### 2.11 Immunofluorescence staining and image analysis

Frozen tissue sections were rinsed three times in PBS. The sections were permeabilised with 0.1% Triton X‐100 in PBS for 20 min, washed three times, and blocked with 10% donkey serum albumin (Biological Industries) for 30 min. Then sections were processed for immunofluorescence using 6E10 (1:500, mouse, 803014, BioLegend) antibodies incubated at 4°C overnight, followed by secondary antibodies conjugated to Alexa Fluor 488 (1:400, donkey, A21202, Thermo). The sections were coated with an antifade mounting medium containing DAPI and observed under a fluorescence microscope.

### 2.12 Beta secretase activity assay

The beta‐secretase activity assay was performed as previously described (Darnell Jr., 2002). The activity of BACE1 was detected using a beta‐secretase activity assay kit (Abcam) and measured using a microplate reader with a multi‐wavelength measurement system with Ex/Em = 335/495 nm.

### 2.13 Chromatin immunoprecipitation assay

Chromatin immunoprecipitation (ChIP) assays were performed in brain tissues and cells using antibodies against MEIS2 according to a modified CST protocol (https://www.cellsignal.cn/learn‐and‐support/protocols/protocol‐chip‐agarose). The samples were cross‐linked in 1% formaldehyde (Thermo) for 10–15 min and terminated with 125 mM glycine (Sangon Biotech) for 5 min. The nucleus was extracted, and DNA fragments of 150–900 bp were used for subsequent steps. Chromatin solution was immunoprecipitated by MEIS2 (1:100, mouse, sc‐515470, Santa Cruz) or IgG antibodies (1:100, mouse, 10400C, Thermo Fisher) overnight at 4°C. Protein A/G Agarose Beads (Thermo) were used to adsorb the DNA‐antibody complex; following this, the input and immunoprecipitated samples were treated with proteinase K for 90 min at 60°C. DNA was purified using a DNA purification kit (Beyotime) and analysed using RT‐qPCR. Primer sequences are listed in Table S5.

### 2.14 Luciferase assay

To determine the promoter activity, plasmids containing all of the MEIS2‐binding sites on *Bace1* promoter (pGL4.1‐BACE1) bearing 2100 bp (−2000 to +100 from the transcription start site), the mutation constructs with mutations at −1955 bp to −1940 bp (Site 1), −1480 bp to −1465 bp (Site 2), −1451 bp to −1436 bp (Site 3), −376 bp to −355 bp (Site 4) or + 20 bp to +35 bp (Site 5) and the empty plasmid pGL4.1 (a negative control) were co‐transfected with pcDNA3.1‐MEIS2 or pcDNA3.1 vector into N2a cells using Lip2000 transfection reagent (Invitrogen) following the manufacturer's instructions. Forty‐eight hours after transfection, cells were collected and lysed. Firefly and Renilla luciferase activities were measured using a luciferase assay kit (Beyotime).

### 2.15 Actinomycin D assay

The actinomycin D assay was performed to stop transcription. After 24 h of plasmid transfection, the HT22 cells were treated with 5 μg/mL actinomycin D (MCE) for 3 h. mRNA levels were detected by RT‐qPCR and normalised to β‐actin levels.

### 2.16 RNA‐sequencing

Total RNA extraction using TRIzol reagent (Invitrogen) and RNA integrity testing using Agilent 2100 bioanalyzer. The mRNA enrichment process involved utilizing Oligo (dT) magnetic beads to select for transcripts with polyadenylated tails. The obtained mRNA is subjected to random fragmentation using divalent cations within the NEB Fragmentation Buffer. Subsequent library construction followed the NEB standard protocol. Upon completion of library construction, the libraries were initially quantified using the Qubit 2.0 Fluorometer, and then diluted to a concentration of 1.5 ng/μL. Subsequently, the insert size of the libraries was assessed using the Agilent 2100 bioanalyzer. Following confirmation that the insert size was qualified, qRT‐PCR was employed to accurately quantify the effective concentration of the libraries (ensuring an effective concentration exceeding 1.5 nM) to ensure library quality. Then different libraries were pooled according to their effective concentrations and the desired amount of data for sequencing on the Illumina platform. DESeq2 was performed for differential gene expression (DEGs) analysis.

### 2.17 KEGG, GO and Reactome enrichment

Conduct Kyoto Encyclopedia of Genes and Genomes (KEGG) pathway enrichment analysis, gene ontology (GO) functional enrichment analysis, and Reactome functional enrichment analysis on the sets of differentially expressed genes using the clusterProfiler software. The *p* < 0.05 is considered significant.

### 2.18 Statistical analyses

All data are expressed as the mean ± standard error of the mean (SEM). Unpaired two‐tailed Student's *t*‐test, one‐way analysis of variance (ANOVA) with Tukey's test and simple linear regression were performed to compare data. GraphPad (GraphPad Prism software) was used for statistical analysis. Differences were considered statistically significant at *p* < 0.05.

## 3 RESULTS

### 3.1 MEIS2 increases in AD

Studies have reported that MEIS2 is related to mental retardation and neurodegenerative diseases (Huang et al., 2019; Liu et al., 2020). To clarify the role of MEIS2 in AD, we first retrieved the levels of MEIS2 from the AlzData database. The result showed an upregulation tendency in the hippocampus and temporal cortex of AD patients (Figure S1) (Xu et al., 2018).

**FIGURE 1:**
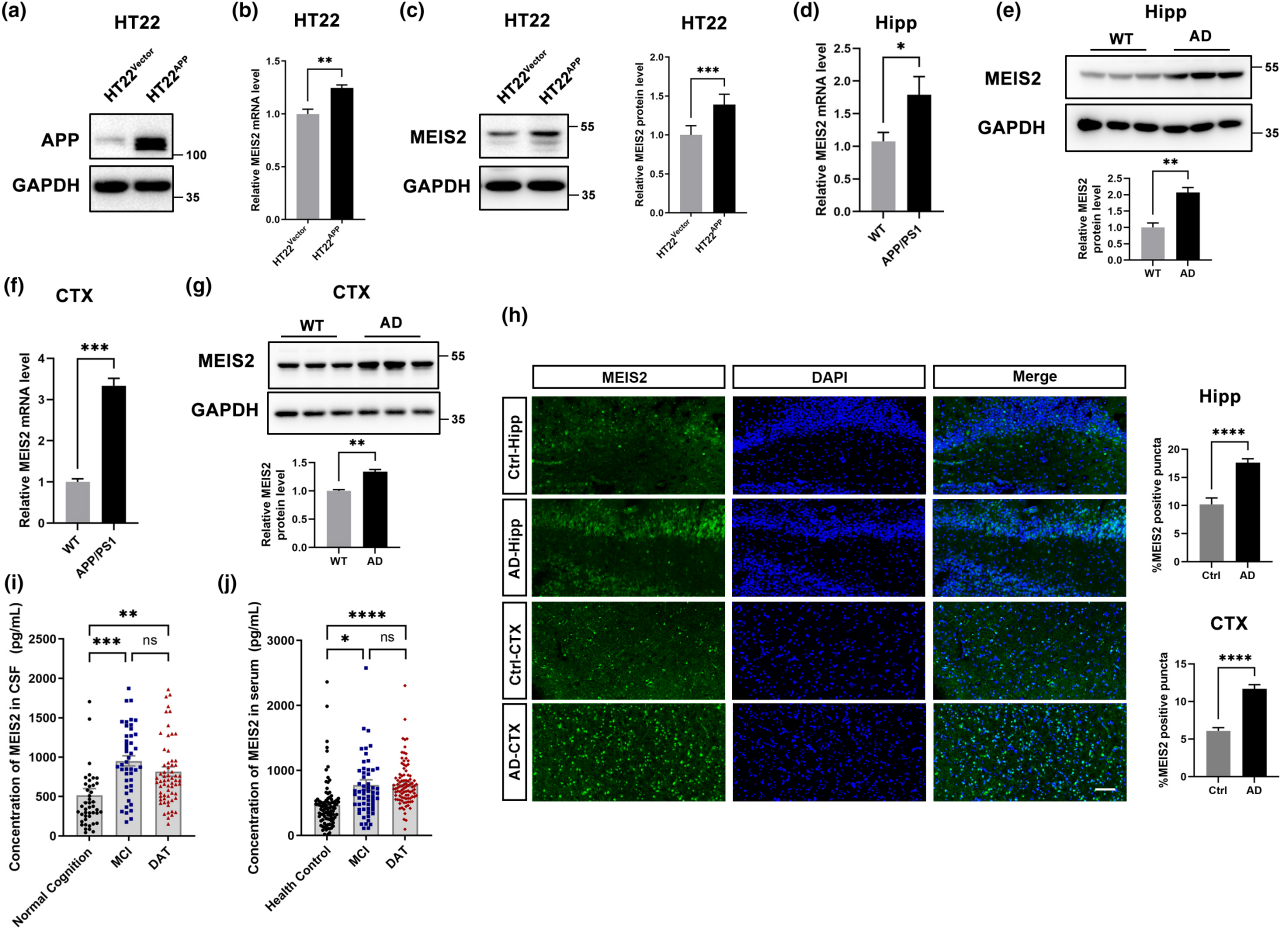
MEIS2 increases in AD. (a) Immunoblot analysis of APP protein levels in HT22 cells transfected with APP_swe_ or the control vector. (b) MEIS2 mRNA levels in HT22 cells transfected with APP_swe_ or the control vector. (c) Representative western blots and relative quantification of the protein expression levels of MEIS2 in HT22 cells transfected with APP_swe_ or the control vector. (d) MEIS2 mRNA levels in the hippocampus of 8‐month APP/PS1 mice and WT mice (*n* = 3). (e) MEIS2 protein levels in the hippocampus of 8‐month APP/PS1 mice and WT mice (*n* = 3). (f) MEIS2 mRNA levels in the cortex of 8‐month APP/PS1 mice and WT mice (*n* = 3). (g) MEIS2 protein levels in the cortex of 8‐month‐old APP/PS1 and WT mice (*n* = 3). (h) Representative immunostaining and quantifications of MEIS2 (green) and DAPI in the hippocampus and cortex of patients with AD (*n* = 3) and control cases (*n* = 4). Scale bar = 100 μm. (i) MEIS2 levels in the CSF of MCI (*n* = 45) and DAT (*n* = 65) of patients with AD and normal cognition group (*n* = 45). (j) MEIS2 levels in the sera of AD patients with mild cognitive impairment (MCI) (*n* = 60), dementia stage of AD (DAT) (*n* = 95) and participants with normal cognition (*n* = 95). Data are presented as mean ± standard error of the mean (SEM) of three separate experiments. (*p < 0.05, **p < 0.01, ***p < 0.001, ****p < 0.0001). Data in b–h are analysed by Student's *t*‐test; data in i and j were analysed by one‐way ANOVA.

Subsequently, we cultured HT22 cells stably expressing APP_Swe_ to mimic familial AD (Figure 1a).

The cells were collected to examine the mRNA (Figure 1b) and protein (Figure 1c) levels of MEIS2. The results showed that MEIS2 was markedly elevated in HT22^APP^ cells. Then we tested the mRNA (Figure 1d) and protein (Figure 1e) levels of MEIS2 in the hippocampus of 8‐month‐old APP/PS1 double‐transgenic mice and WT mice. MEIS2 levels were significantly increased in APP/PS1 mice compared with WT mice. Similar results were observed in the cortical tissues of APP/PS1 mice (Figure 1f,g). After testing MEIS2 levels in the hippocampus and temporal cortex of AD and age‐matched control patients (characteristics of the enrolled cases are shown in Table S1), we found MEIS2 positive puncta were significantly increased in the AD group (Figure 1h).

We then investigated alterations in MEIS2 levels in the body fluids of AD patients. Human CSF and serum samples were collected. The demographic characteristics of enrolled patients are presented in Tables S2 and S3. In CSF samples, MEIS2 levels were increased in the mild cognitive impairment (MCI) and the dementia stage of Alzheimer's disease (DAT). The average MEIS2 levels in MCI‐stage (953.30 ± 64.45 pg/mL) and DAT‐stage (816.90 ± 59.55 pg/mL) were 1.85‐ and 1.59‐fold higher than that in the normal cognition (NC) group (Figure 1i). Serum MEIS2 levels were also increased in MCI‐stage (776.10 ± 81.67 pg/mL) and DAT‐stage (788.90 ± 34.07 pg/mL), which were 1.65‐ and 1.68‐fold higher than in the HC group (Figure 1j) respectively.

These data showed that MEIS2 increased in AD, and MEIS2 might play a critical role in AD.

### 3.2 MEIS2 increases with age and has a strong correlation with BACE1 and amyloid cleavage products in a vivo model of Alzheimer's disease

To investigate the function of MEIS2 during the course of AD, we explored whether the difference in MEIS2 expression between the AD model and WT groups was age‐dependent and associated with BACE1 levels.

**FIGURE 2:**
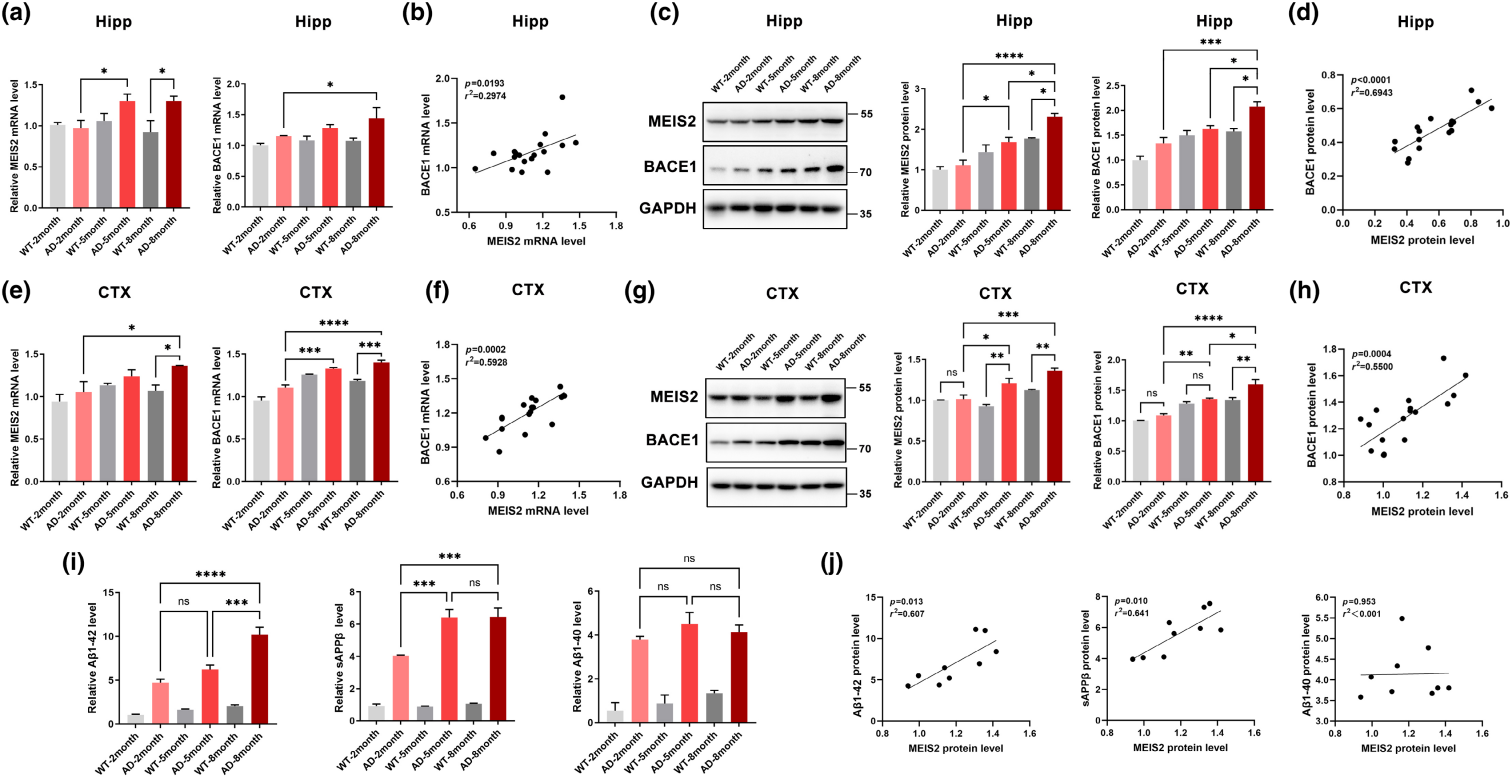
MEIS2 increases with age in APP/PS1 mice and has a strong correlation with BACE1 and amyloid cleavage products. (a) MEIS2 and BACE1 mRNA levels in the hippocampi of 2‐, 5‐, and 8‐month APP/PS1 mice and age‐matched WT mice. (b) Correlation between MEIS2 and BACE1 mRNA levels in mice hippocampi. (c) Representative western blots and relative quantification of the protein expression levels of MEIS2 and BACE1 in the hippocampi of 2‐, 5‐, and 8‐month APP/PS1 mice and age‐matched WT mice. (d) Correlation between MEIS2 and BACE1 protein levels in mice hippocampi. (e) MEIS2 and BACE1 mRNA levels in the cortices of 2‐, 5‐, and 8‐month APP/PS1 mice and age‐matched WT mice. (f) Correlation between MEIS2 and BACE1 mRNA levels in mice cortices. (g) Representative western blots and relative quantification of the protein expression levels of MEIS2 and BACE1 in the cortices of 2‐, 5‐, and 8‐month APP/PS1 mice and age‐matched WT mice. (h) Correlation between MEIS2 and BACE1 protein levels in mice cortices. (i) The relative levels of soluble Aβ_1‐42_, sAPPβ, and Aβ_1‐40_ in the cortices of 2‐, 5‐, and 8‐month APP/PS1 mice and age‐matched WT mice were detected by ELISA. (j) Correlation analysis of Aβ_1‐42_, sAPPβ, and Aβ_1‐40_ levels with MEIS2 expression in the cortices of APP/PS1 mice at different ages. Male APP/PS1 mice of 2, 5, and 8 months (AD group, *n* = 3 for each group) and male age‐matched WT mice (WT group, *n* = 3 for each group) are used. Data are presented as mean ± SEM. (*p < 0.05, **p < 0.01, ***p < 0.001, ****p < 0.0001). Data in a, c, e, g, and i are analysed by one‐way ANOVA; data in b, d, f, h, and j are analysed by linear regression analysis.

We first analysed the mRNA and protein levels of MEIS2 and BACE1 in the hippocampal tissues at different ages of APP/PS1 mice and age‐matched controls. During the period of 2‐ to 5‐ months old, the mRNA level of MEIS2 in the hippocampus of APP/PS1 mice significantly increased, and the mRNA expression of BACE1 in the hippocampus of APP/PS1 mice at 8 months old was significantly higher than that of 2 months old (Figure 2a). The MEIS2 and BACE1 protein levels were also markedly increased in the 8‐month APP/PS1 mice, compared with 2‐ or 5‐month APP/PS1 mice or the 8‐month WT group (Figure 2c). Next, we performed a simple linear regression and found a positive correlation between the mRNA and protein levels of MEIS2 and BACE1 (Figure 2b,d); similar results were observed in cortical tissues (Figure 2e–h), indicating that the increased expression of BACE1 in APPPS1 mice might be related to MEIS2.

As APP is initially cleaved by BACE1 in AD to generate Aβ and sAPPβ, we wished to confirm whether the levels of amyloid cleavage products were consistent with MEIS2 expression. We measured the levels of soluble Aβ_1‐42_, sAPPβ, and Aβ_1‐40_ in the brain tissues of the AD model and WT mice with different ages (Figure 2i) and observed that MEIS2 levels were positively correlated with the expression of Aβ_1‐42_ and sAPPβ (Figure 2j).

MEIS2 levels were elevated with the progression of AD and strongly related to BACE1 levels, as well as the levels of amyloid cleavage products in an in vivo model. Thus, we speculate that MEIS2 may play a critical role in the process of AD via regulating BACE1.

### 3.3 MEIS2 promotes APP amyloid cleavage via upregulating BACE1 in vitro

**FIGURE 3:**
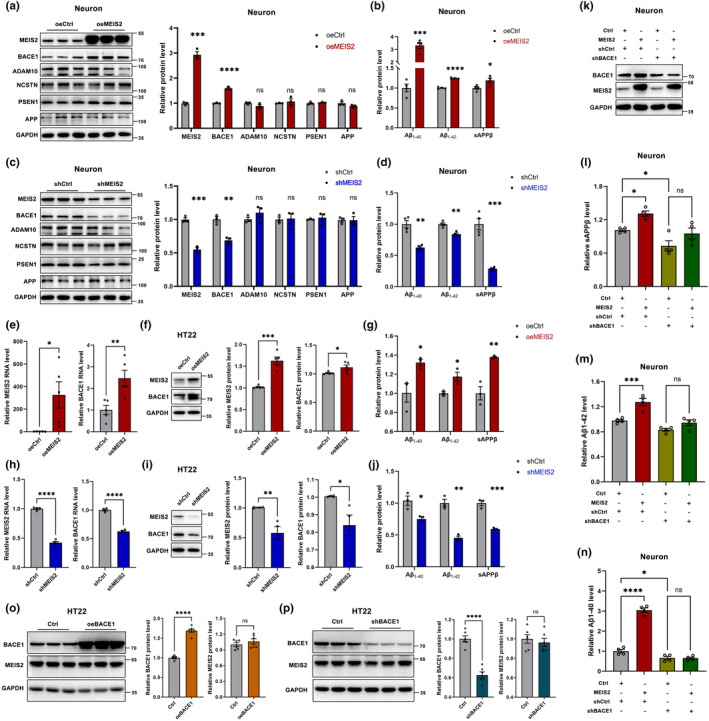
The effect of MEIS2 on BACE1 expression and APP amyloid cleavage. (a) Representative western blots and relative quantification of the protein expression levels of MEIS2, BACE1, ADAM10, NCSTN, PSEN1, and APP in mouse primary neurons transfected with oeMEIS2 or the control vector. (b) The relative levels of Aβ_1‐40_, Aβ_1‐42_, and sAPPβ in the culture media of mouse primary neurons transfected with oeMEIS2 or the control vector. (c) Representative western blots and relative quantification of the protein expression levels of MEIS2, BACE1, ADAM10, NCSTN, PSEN1, and APP in mouse primary neurons transfected with MEIS2‐shRNA or the control‐shRNA. (d) The relative levels of Aβ_1‐40_, Aβ_1‐42_, and sAPPβ in the culture medium of mouse primary neurons transfected with MEIS2‐shRNA or the control‐shRNA. (e) MEIS2 and BACE1 mRNA levels in HT22 cells transfected with MEIS2 plasmid or control vector. (f) MEIS2 and BACE1 protein levels in HT22 cells transfected with MEIS2 plasmid or control vector. (g) The relative levels of Aβ_1‐40_, Aβ_1‐42_, and sAPPβ in the culture medium of HT22 cells transfected with MEIS2 plasmid or control vector. (h) MEIS2 and BACE1 mRNA levels in HT22 cells transfected with LV_shMEIS2_ or LV_shCtrl_. (i) MEIS2 and BACE1 protein levels in HT22 cells transfected with LV_shMEIS2_ or LV_shCtrl_. (j) The relative levels of Aβ_1‐40_, Aβ_1‐42_, and sAPPβ in the culture medium of HT22 cells transfected with LV_shMEIS2_ or LV_shCtrl_. (k) MEIS2 and BACE1 in primary mouse neurons co‐transfected with oeMEIS2 or the control vector and LV_shBACE1_ or LV_shCtrl_, respectively. The relative levels of sAPPβ (l), Aβ_1‐42_ (m), and Aβ_1‐40_ (n) in the culture medium of mouse primary neurons co‐transfected with oeMEIS2 or the control vector and LV_shBACE1_ or LV_shCtrl_, respectively. (o) BACE1 and MEIS2 protein levels in HT22 cells transfected with BACE1 plasmid and control vector. (p) BACE1 and MEIS2 protein levels in HT22 cells transfected with shBACE1 plasmid and control vector. Data are presented as mean ± SEM of three separate experiments. (*p < 0.05, **p < 0.01, ***p < 0.001, ****p < 0.0001). Data in a–j and o–p are analysed by Student's *t*‐test; data in l–n are analysed by one‐way ANOVA.

Considering the association among MEIS2, BACE1, and APP amyloid degradation products, we further explored the regulatory relationship among the three in vitro. Assessing MEIS2 and BACE1 levels in primary mouse neurons overexpressing MEIS2, the results showed MEIS2 upregulation markedly increased BACE1 protein levels (Figure 3a). Meanwhile, the high expression of MEIS2 cannot affect the level of Nicastrin (NCSTN) and Presenilin 1 (PSEN1), which are the main components of the γ‐secretase complex (Esler et al., 2002; Yu et al., 2000). The full‐length APP and the α‐secretase of APP, A disintegrin and metalloproteinase 10 (ADAM10) were still not detected differential expression.

The soluble Aβ_1‐40_, Aβ_1‐42_, and sAPPβ (products of APP amyloid cleavage) were significantly elevated after MEIS2 overexpression (Figure 3b), indicating that MEIS2 could affect the process of amyloid deposition. In addition, MEIS2 knockdown reduced BACE1, Aβ_1‐40_, Aβ_1‐42_, and sAPPβ in primary neuron cells (Figure 3c,d). The similar results were also investigated in HT22 cells (Figure 3e–j).

To further demonstrate whether MEIS2 promotes amyloidogenic cleavage of APP depends on upregulating BACE1, primary mouse neurons were co‐transfected with the oeMEIS2 vector and BACE1‐shRNA (Figure 3k). MEIS2 overexpression elevated the levels of APP amyloid cleavage products in the culture medium; in contrast, BACE1 silencing blocked the upregulation of sAPPβ, Aβ_1‐42_, and Aβ_1‐40_ increased by MEIS2 upregulation (Figure 3l,m,n).

Notably, overexpression or downregulation of BACE1 did not affect the protein levels of MEIS2 in a short period, suggesting that MEIS2 acts upstream of BACE1 (Figure 3o,p).

### 3.4 MEIS2 upregulation aggravates cognitive impairment, elevates BACE1 expression, and promotes APP amyloid cleavage in vivo

**FIGURE 4:**
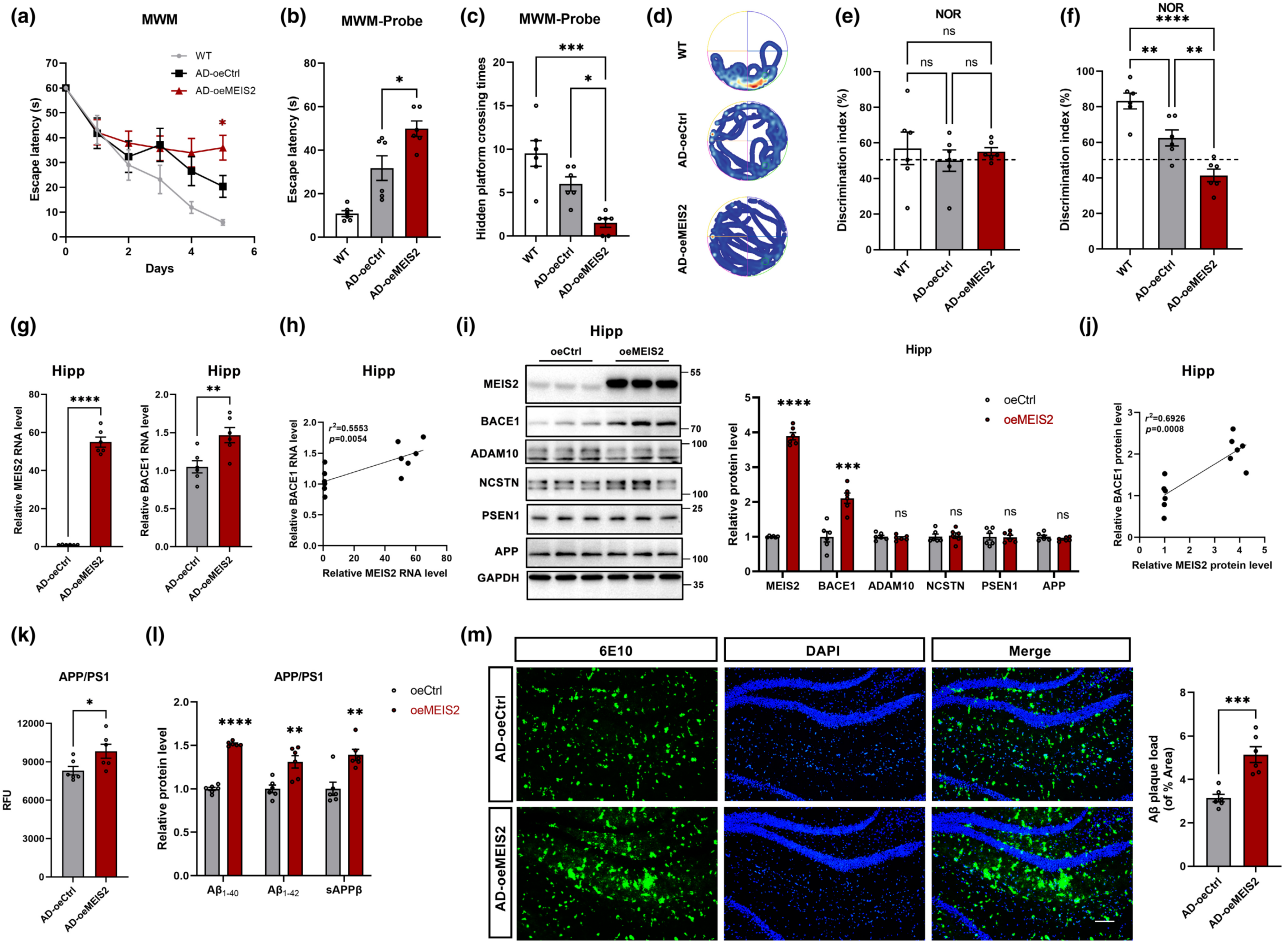
Aggravated cognitive impairment, upregulated BACE1 expression, and promoted amyloid cleavage in the APP/PS1 mice injected with AAV_oeMEIS2_. APP/PS1 mice were microinjected with AAV_oeMEIS2_ or AAV_oeCtrl_ (3 × 10^9^ viral genomes/site). (a–f) WT mice (WT, *n* = 6), AAV_oeMEIS2_‐injected APP/PS1 mice (AD‐oeMEIS2, *n* = 6), and AAV_oeCtrl_‐injected APP/PS1 mice (AD‐oeCtrl, *n* = 6) were trained and tested for behavioural experiments. (a) Morris water maze (MWM) test results depicting latency to escape to a hidden platform in the 5‐day training phase of the APP/PS1 mice injected with AAV_oeMEIS2_. MWM probe test to analyse (b) the latency to escape to the platform location and (c) the times of AAV_oeMEIS2_‐injected APP/PS1 mice passed through the platform location. (d) Representative tracking heat map of probe test of the AAV_oeMEIS2_‐injected APP/PS1 mice. Novel object recognition analysed the discrimination index during training (e) and testing (f) of the APP/PS1 mice injected with AAV_oeMEIS2_. (g) The mRNA levels of MEIS2 and BACE1 in the cerebral hippocampi of APP/PS1 mice injected with AAV_oeCtrl_ and AAV_oeMEIS2_. (h) Correlation between MEIS2 and BACE1 mRNA levels in mice hippocampi. (i) Representative Western blots and relative quantification of the protein expression levels of MEIS2, BACE1, ADAM10, NCSTN, PSEN1, and APP in the cerebral hippocampi of APP/PS1 mice injected with AAV_oeCtrl_ and AAV_oeMEIS2_. (j) Correlation between MEIS2 and BACE1 protein levels in mice hippocampi. (k) Fluorogenic BACE1 activity assay analysis of BACE1 activity in the brains of APP/PS1 mice injected with AAV_oeCtrl_ and AAV_oeMEIS2_. (l) The relative levels of Aβ_1‐40_, Aβ_1‐42_, and sAPPβ in the brain tissues of the APP/PS1 mice injected with AAV_oeMEIS2_. (m) Representative immunostaining and quantification of 6E10‐positive amyloid plaques in the brains of the APP/PS1 mice injected with AAV_oeMEIS2_. Scale bar = 100 μm. Data are presented as mean ± SEM. (*p < 0.05, **p < 0.01, ***p < 0.001, ****p < 0.0001). Data in a, b, c, e, and f are analysed by one‐way ANOVA; data in g, i, k, l, and m are analysed by Student's *t*‐test; data in h and j are analysed by linear regression analysis.

To explore the effect of MEIS2 in AD model mice, we generated adeno‐associated viral vectors (AAV) carrying the MEIS2 cDNA (AAV_oeMEIS2_) and MEIS2 shRNA (AAV_shMEIS2_). Viruses were microinjected into the hippocampus by stereotactic technology. APP/PS1 mice were treated with AAV_oeMEIS2_ or AAV_shMEIS2_ at 6 months of age, and their behaviour was examined after 1 month. First, the Morris water maze (MWM) test was employed to assess the cognitive capacity of mice treated with AAV_oeMEIS2_. Compared with the APP/PS1 mice treated with AAV_oeCtrl_, the mice treated with AAV_oeMEIS2_ displayed significantly damaged spatial learning with a longer escape latency to find the hidden platform (Figure 4a). When the platform was removed during the probe trial, AAV_oeMEIS2_‐treated mice spent more time crossing the original position of the platform and had fewer entries into the target site than the AAV_oeCtrl_‐treated mice (Figure 4b,c). According to the representative tracking heat map, we found the MEIS2‐upregulated mice exhibited a chaotic and disorderly swimming trajectory (Figure 4d). The NOR tasks showed no significant difference in the discrimination index between two identical objects in each group of mice during the training period (Figure 4e). However, in the test phase, AAV_oeMEIS2_‐treated mice displayed a lower object recognition index for new objects than AAV_oeCtrl_‐treated mice (Figure 4f). The open‐field behaviour test showed that, compared with WT mice, the traveling distance and time spent in the central area were markedly reduced in APP/PS1 mice, with no significant statistical difference observed in the motor ability of mice. These results indicate that the AD group showed more severe anxiety. However, MEIS2 did not significantly affect athletic ability or anxiety in the APP/PS1 mice (Figure S3A–C).

Then we analysed BACE1 expression in hippocampal sections. The mRNA (Figure 4g) and protein (Figure 4i) levels of BACE1 were enhanced significantly in the AAV_oeMEIS2_‐treated group, and the β‐secretase activity was also elevated (Figure 4k). Meanwhile, the high expression of MEIS2 cannot affect the NCSTN, PSEN1, ADAM10, and APP levels, which indicated MEIS2 upregulation markedly increased BACE1 protein levels instead of α‐ or γ‐ secretase (Figure 4i). Moreover, there was a significant correlation between BACE1 and MEIS2 expression when MEIS2 was upregulated in mouse brain tissue after AAV_oeMEIS2_ injection (Figure 4h,j).

To analyse the influence of MEIS2 on APP amyloid cleavage and Aβ deposition, we measured the levels of soluble Aβ_1‐40_, Aβ_1‐42_, and sAPPβ in mouse brain lysates using ELISA. The critical products of the APP amyloidogenic cleavage pathway were markedly elevated in AAV_oeMEIS2_‐infected APP/PS1 mice compared with AAV_oeCtrl_‐infected APP/PS1 mice. (Figure 4l). Subsequently, we stained Aβ plaques by immunofluorescence. The number of Aβ plaques, which are the pathological hallmark of AD, was markedly increased in the MEIS2‐overexpressed APP/PS1 mice (Figure 4m).

These data suggest that MEIS2 overexpression aggravated cognitive impairment in the APP/PS1 mice; this might result from BACE1 upregulation and promotion of APP amyloid cleavage.

### 3.5 Deficiency of MEIS2 alleviates cognitive impairment and reduces BACE1 expression and APP amyloid cleavage in APP/PS1 mice

**FIGURE 5:**
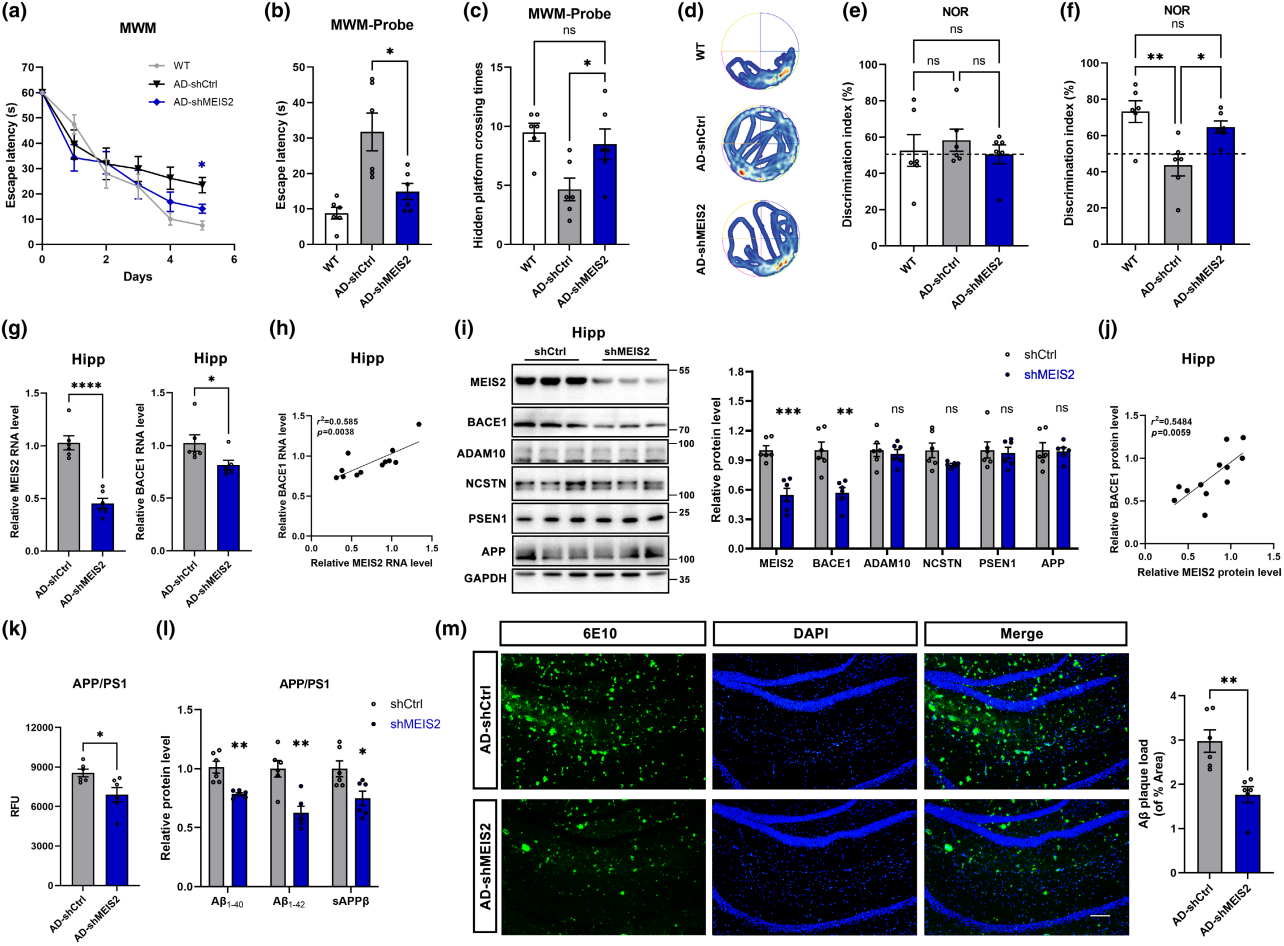
Alleviated cognitive impairment, downregulated BACE1 expression, and reduced amyloid cleavage in the APP/PS1 mice injected with AAV_shMEIS2_. APP/PS1 mice were microinjected with AAV_shMEIS2_ or AAV_shCtrl_ (3 × 10^9^ viral genomes/site). (a–f) WT mice (WT, *n* = 6), AAV_shMEIS2_‐injected APP/PS1 mice (AD‐shMEIS2, *n* = 6), and AAV_shCtrl_‐injected APP/PS1 mice (AD‐shCtrl, *n* = 6) were trained and tested for behavioural experiments. (a) MWM test results depicting latency to escape to a hidden platform in the 5‐day training phase of the APP/PS1 mice injected with AAV_shMEIS2_. MWM probe test to analyse (b) the latency to escape to the platform location and (c) the times of AAV_shMEIS2_‐injected APP/PS1 mice passed through the platform location. (d) Representative tracking heat map of probe test of the AAV_shMEIS2_‐injected APP/PS1 mice. Novel object recognition analysed the discrimination index during training (e) and testing (f) of the APP/PS1 mice injected with AAV_shMEIS2_. (g) The mRNA levels of MEIS2 and BACE1 in the cerebral hippocampi of APP/PS1 mice injected with AAV_shCtrl_ and AAV_shMEIS2_. (h) Correlation between MEIS2 and BACE1 mRNA levels in mice hippocampi. (i) Representative Western blots and relative quantification of the protein expression levels of MEIS2, BACE1, ADAM10, NCSTN, PSEN1 and APP in the cerebral hippocampi of APP/PS1 mice injected with AAV_shCtrl_ and AAV_shMEIS2_. (j) Correlation between MEIS2 and BACE1 protein levels in mice hippocampi. (k) Fluorogenic BACE1 activity assay analysis of BACE1 activity in the brains of APP/PS1 mice injected with AAV_shCtrl_ and AAV_shMEIS2_. (l) The relative levels of Aβ_1‐40_, Aβ_1‐42_, and sAPPβ in the brain tissues of the APP/PS1 mice injected with AAV_shMEIS2_. (m) Representative immunostaining and quantification of 6E10‐positive amyloid plaques in the brains of the APP/PS1 mice injected with AAV_shMEIS2_. Scale bar = 100 μm. Data were presented as mean ± SEM. (*p < 0.05, **p < 0.01, ***p < 0.001, ****p < 0.0001). Data in a, b, c, e, and f are analysed by one‐way ANOVA; data in g, i, k, l, and m are analysed by Student's *t*‐test; data in h and j are analysed by linear regression analysis.

To further elucidate whether MEIS2 deficiency could reduce BACE1 expression and alleviate Aβ pathology‐associated cognitive deficits in AD, we tested the learning and memory ability of APP/PS1 mice treated with AAV_shMEIS2_. Compared with the control group, mice with MEIS2 downregulation displayed improved learning capacity, as indicated by decreased escape latency during the 5‐day training phase (Figure 5a), without affecting the motor ability or anxiety state of AD animals (Figure S3D–F).

Moreover, in the probe test, mice treated with AAV_shMEIS2_ spent significantly less time entering the target area (Figure 5b) and more times crossing the platform location than control mice (Figure 5c). The representative tracking heat map of the probe test supports these results (Figure 5d). In the NOR test, each group showed similar cognitive index for the same object (Figure 5e). The object recognition index of MEIS2‐downregulated mice was remarkably higher than that of AAV_shCtrl_‐treated APP/PS1 mice (Figure 5f).

We demonstrated that mRNA and protein levels of BACE1 and β‐secretase activity were reduced in AAV_shMEIS2_‐treated mice with no significant difference detected in APP, ADAM10, PSEN1, and NCSTN (Figure 5g, i,k). A positive correlation between MEIS2 and BACE1 was confirmed in both mRNA and protein levels when MEIS2 was knocked down by AAV_shMEIS2_‐injection (Figure 5h,j).

APP cleavage and Aβ synthesis were examined among the groups. We found that the levels of soluble Aβ_1‐40_, Aβ_1‐42_, and sAPPβ were significantly lower in the brain of AAV_shMEIS2_‐treated APP/PS1 mice compared with AAV_shCtrl_‐treated mice (Figure 5l). Downregulation of MEIS2 by AAV_shMEIS2_ reduced the number of amyloid plaques in the brains of APP/PS1 mice (Figure 5m).

Therefore, MEIS2 inhibition could alleviate cognitive impairment, BACE1 expression, and APP amyloid cleavage in 6‐month APP/PS1 mice, suggesting that it could be a potential target for early intervention in AD.

### 3.6 MEIS2 is a novel transcriptional factor of BACE1

**FIGURE 6:**
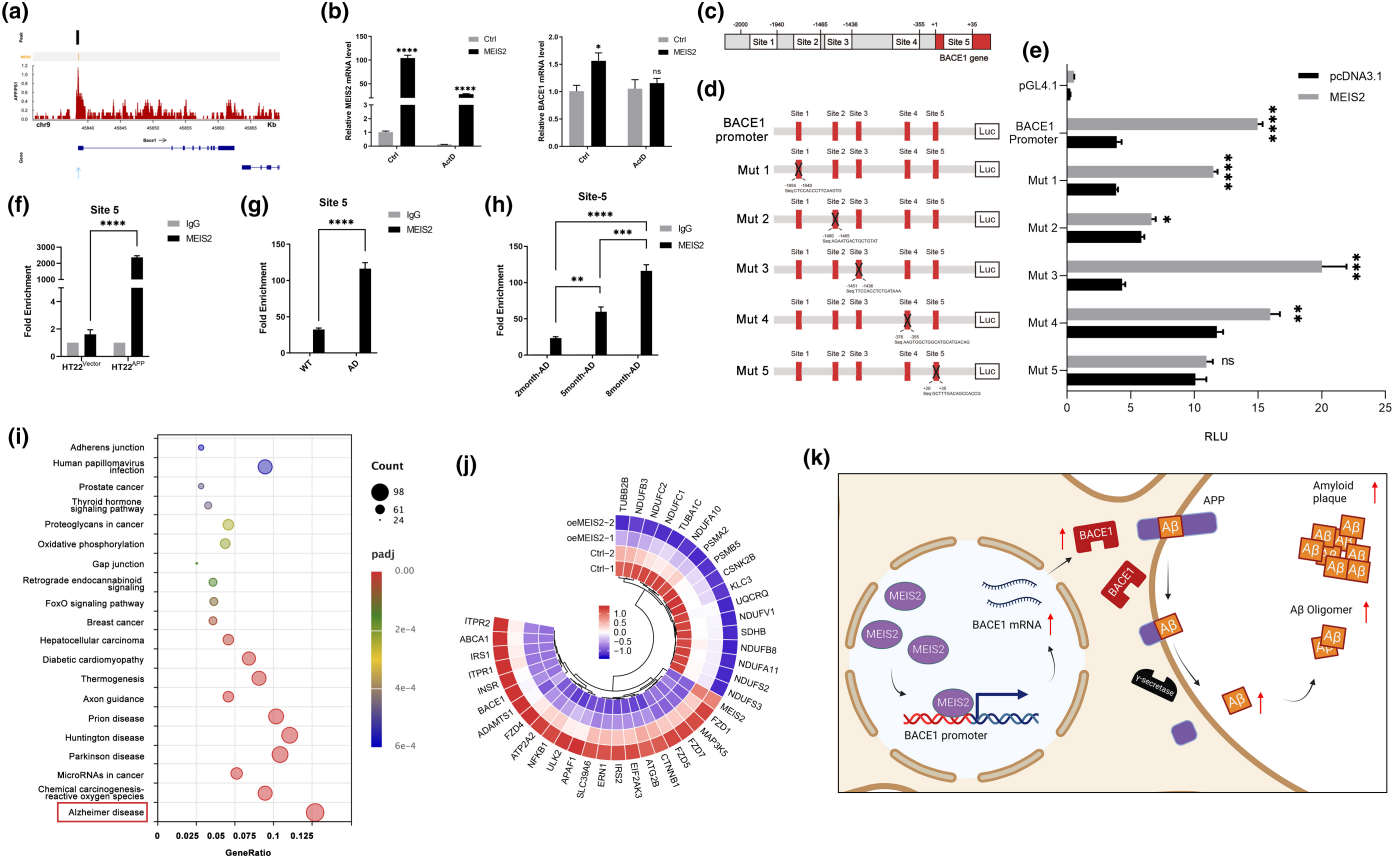
MEIS2 binds to BACE1 promoter and regulates BACE1. (a) Integrative Genomics Viewer (IGV) of the chromatin‐accessible regions of BACE1 was analysed by GSE145908 in the GEO database. (b) The mRNA levels of MEIS2 and BACE1 in HT22 cells treated with 5 μg/mL ActD for 3 h after transfected with MEIS2 plasmid and control vector for 24 h. (c) The potential MEIS2 binding sites on mouse BACE1 promoter were predicted by the JASPAR database. (d) Schematic diagram of the mutation constructs of MEIS2 binding sites in the mouse BACE1 promoter region. (e) Dual‐luciferase reporter assay results depicted BACE1 promoter activity in N2a cells after being co‐transfected with MEIS2 or pcDNA3.1 and mouse BACE1 promoter luciferase reporter plasmid (−2000 to TSS) or different mutations with renilla reporter plasmid as the control. The ChIP assay results depicted MEIS2 binding to the BACE1 promoter in HT22^APP^ cells (f), the brain of 8‐month age APP/PS1 mice and age‐matched WT mice (g), and the APP/PS1 mice with different ages (h). (i) KEGG pathway analysis of DEGs after RNA‐seq using HT22 cells transfected with MEIS2 plasmid and control vector. (j) Circular heatmap of AD‐related DEGs after transfected with MEIS2 plasmid and control vector in HT22 cells. (k) Diagram showing the mechanism by which MEIS2 promotes amyloid cleavage of β amyloid precursor protein through transcriptional regulation of BACE1. Data are presented as mean ± SEM of three separate experiments. (*p < 0.05, **p < 0.01, ***p < 0.001, ****p < 0.0001). Data in b, e, f and g are analysed by Student's *t*‐test; data in h is analysed by one‐way ANOVA.

As MEIS2 is involved in transcription regulation (Dupacova et al., 2021; Zha et al., 2014) and our sequencing revealed a chromatin‐accessible region in the putative promoter region of *Bace1* (ATAC‐seq) that contained the monomeric binding motif ‘TGACAG’ of MEIS2 (Figure 6a) (Schulte & Geerts, 2019), we investigated whether MEIS2 directly affected BACE1 through the transcription pathway. We assessed the effect of the transcriptional inhibitor actinomycin D (ActD) on BACE1 mRNA levels in MEIS2‐transfected cells. The results showed that MEIS2 overexpression increased BACE1 mRNA levels in HT22 cells; however, ActD significantly inhibited MEIS2 overexpression‐induced BACE1 upregulation (Figure 6b), indicating that MEIS2 regulates BACE1 expression through the transcriptional pathway.

To further examine the mechanism of MEIS2 regulating BACE1 transcription, we analysed the sequence of *Bace1* promoter in the JASPAR database and found five potential MEIS2‐binding sites (Figure 6c) (Sandelin et al., 2004). The predicted sites were − 1955 bp to −1940 bp (Site 1), −1480 bp to −1465 bp (Site 2), −1451 bp to −1436 bp (Site 3), −376 bp to −355 bp (Site 4) and + 20 bp to +35 bp (Site 5) to the TSS of *Bace1* promoter respectively. We then linked the *Bace1* promoter containing different point mutations to the firefly luciferase reporter gene in the promoter‐less vector pGL4.10‐basic (Figure 6d). N2a cells were co‐transformed with MEIS2 plasmids, promoter reporters, and Renilla luciferase reporters. MEIS2 overexpression was confirmed by western blotting (Figure S4A). The dual‐luciferase reporter assay indicated that MEIS2‐transfected cells showed higher relative luciferase activity than the cells with the empty vector control. Deletion of 15 bp from the +20 to +35 bp region (Site 5) blocked the activation of the *Bace1* promoter (Figure 6e), suggesting that Site 5 was a positive regulatory element in the *Bace1* promoter region.

To determine whether MEIS2 bind to the *Bace1* promoter at Site 5, we performed chromatin immunoprecipitation (ChIP) experiments with HT22 cells stably expressing the APP gene and the empty vector control and observed that MEIS2 was significantly enriched at Site 5 compared with the control group (Figure 6f). In the brains of the APP/PS1 transgenic mice, the enrichment level of MEIS2 at site 5 in the *Bace1* promoter was significantly higher than that in WT mice (Figure 6g) and increased remarkably with age in the AD model (Figure 6h). These findings suggest that MEIS2 binds to the *Bace1* promoter to regulate BACE1 expression via the transcription pathway.

Considering the wide range of transcription factor functions, we further explored whether MEIS2 affects AD through other pathways. HT22 cells overexpressing MEIS2 were subjected to RNA‐seq. Differentially expressed genes (DEGs) were analyzed by KEGG. The results showed that MEIS2 overexpression mainly affects AD. In addition, MEIS2 also plays an important role in other neurodegenerative diseases such as Parkinson disease and Huntington disease. The role of MEIS2 in miRNA and oxidative phosphorylation might be worth exploring (Figure 6i). Further investigation into the key genes influenced by MEIS2 in AD revealed that, in addition to BACE1, MEIS2 also regulates the expression of AD‐related genes, including ABCA1, ERN1, IRS2, and NFKB1. This suggests that MEIS2 could affect AD through multiple pathways (Figure 6j). GO and Reactome analysis indicate that MEIS2 is associated with mitochondrial function, respiratory electron transport, neuron differentiation, NOTCH1 pathway, and MAPK pathway (Figure S5).

In general, we demonstrated that MEIS2 promoted the amyloid cleavage of APP and the deposition of amyloid plaques by upregulating BACE1 expression, thus aggravating the course of AD (Figure 6k).

## 4 DISCUSSION

Meis2 directly binds to the *Zfp503* and *Six3* promoters to regulate spiny neuron differentiation (Su et al., 2022), which is involved in neuronal conversion from mouse microglia (Matsuda et al., 2019) and maintains striatal neuron survival (Yang et al., 2021). Abnormal expression of MEIS2 has an appreciable effect on neuroblastoma cell proliferation, anchorage‐independent growth and tumorigenicity (Zha et al., 2014). In Lowe syndrome, characterised by intellectual disability, MEIS2 is markedly enriched in induced pluripotent stem cells (Liu et al., 2020). At present, research on MEIS2 in the nervous system is limited to its role in the early proliferation and differentiation of neurons. However, MEIS2 is also expressed in fully differentiated neurons, and its involvement in biological processes remains unclear.

MEIS2 participates in regulating multiple pathways, such as the NF‐κB signaling pathway (Wen et al., 2021), Notch pathway (Yan et al., 2022), and Wnt/β‐catenin pathway (Guan et al., 2019; Wang et al., 2019), which also play important roles in the pathological mechanism of AD (Kapoor & Nation, 2021; Lian et al., 2015; Macyczko et al., 2023; Ramachandran et al., 2021; Sun et al., 2022; Wang, Huang, et al., 2022). An imaging‐based study showed a significant association of MEIS2 with mental retardation, learning disabilities, and aging, which might impact AD or other neurodegenerative diseases (Huang et al., 2019). However, its relevance to AD has not been fully explored. The transcriptional level of BACE1, the rate‐limiting enzyme for amyloidogenic cleavage of APP to toxic Aβ, is elevated in the brains of AD patients (Bahn et al., 2019; Nowak et al., 2006). We previously found a chromatin‐accessible region in the potential promoter region of *Bace1* (Wang, Zhang, et al., 2020), which contained the conjectural binding motif “TGACAG” of MEIS2 protein (Schulte & Geerts, 2019). This offers us a good opportunity to explore the pathological function of MEIS2 in AD progression.

We first assessed MEIS2 expression in AD cellular models, examining the hippocampus and cortex regions of AD animal models—the APP/PS1 transgenic mice. Furthermore, we evaluated the levels of MEIS2 in the brain tissue, cerebrospinal fluid, and serum samples derived from AD patients. The findings consistently indicated a significant elevation in MEIS2 expression levels across these AD‐relevant contexts (Figure 1). In our AD in vivo models, we also observed a significant correlation among the age‐dependent expression of MEIS2, BACE1 and APP amyloid cleavage products in the brains of APP/PS1 double transgenic mice (Figure 2). Hence, we hypothesized that MEIS2 potentially exerted an influence on AD.

In vitro experimentation provided additional confirmation that MEIS2 significantly influences BACE1 and the amyloid cleavage process of APP. Notably, these effects were blocked by BACE1 shRNA. Furthermore, modulation of BACE1 expression via knockdown or upregulation did not alter MEIS2 expression levels in a short time (Figure 3). In vivo experiments, we found that MEIS2 overexpression promoted BACE1 expression and significantly impaired the cognition of the APP/PS1 mice without influencing exercise ability and anxiety, thereby interfering with the progression of AD (Figure 4).

Based on earlier studies with knockout mouse models, BACE1 is involved in brain amyloidogenesis and Aβ deposition (Hampel et al., 2021; Harrison et al., 2003; Luo et al., 2001). BACE1 plays an important role in treating and preventing AD. BACE1 knockout or inhibiting β‐secretase enzyme activity could notably rescue AD animals from memory deficits (Ghosh & Osswald, 2014; Ohno et al., 2004; Thakker et al., 2015). Inhibition of BACE1 by 50% sufficiently decreases Aβ load by about 20% in transgenic mice (Patel et al., 2022), and preventive reduction of BACE1 activity by 20%–30% for a long time may effectively prevent AD (Hampel et al., 2021). Moreover, unlike embryonic death in the PSEN1 knockout mice, BACE1 null mice are phenotypically normal, indicating that BACE1‐regulation has a certain degree of safety (Ohno et al., 2004). MEIS2 knockdown reduces BACE1 expression, APP amyloid cleavage, and learning and memory deficits in APP/PS1 mice (Figure 5), suggesting a new direction for early treatment of AD. The utilization of transcription factors as drug targets in clinical treatment often presents numerous challenges and complexities. One of the primary challenges is many transcription factors are difficult to match with small molecule inhibitors due to their highly disordered protein structures and lack of well‐defined small molecule binding sites (Darnell Jr., 2002; Liu et al., 2006). The proline‐tyrosine‐proline (PYP) motif of MEIS2 could form hydrophobic pockets (Schulte & Geerts, 2019), providing a basis for developing MEIS2‐targeted small‐molecule drugs. In addition, the method of modulating binding proteins also provides an effective targeting strategy for transcription factors. The Hox region of MEIS2 could bind to lots of proteins (Dupacova et al., 2021), and regulating the activity of its binding proteins may affect the function of MEIS2, which presents another opportunity for intervention. At present, small molecule inhibitors targeting MEIS proteins are being developed (Gronemeyer et al., 2004), which provides great hope for the development of AD drugs targeting MEIS2.

Multiple transcriptional regulatory factors for BACE1 have been identified. KLF5, GATA, YY1, Sp1, and STAT1 act as activators (Bum‐Erdene et al., 2019; Estrada‐Ortiz et al., 2016; Zhao et al., 2015). In contrast, NF‐κB and NRF2 repress *BACE1* gene transcription (Bahn et al., 2019; Girgin & Kocabaş, 2023). We demonstrated that MEIS2 functions as a transcriptional activator that binds at +20 to +35 bp to the TSS region of *Bace1* gene promoter. MEIS2 also negatively regulates the expression of specific genes (Bushweller, 2019), and the underlying mechanism of that regulation is still unclear. One possible mechanism is posttranslational modification of the MEIS2 protein. For example, wild‐type STAT1 upregulates BACE1 expression; when blocking the phosphorylation of the Tyr701 residue in STAT1, *BACE1* promoter is suppressed (Yeh et al., 2013). Changes in MEIS2 binding proteins might also be associated with MEIS2 activity. Further studies are required to address these questions. Although there is a significant correlation between MEIS2 and BACE1 expression in an AD model, the degree of BACE1 expression varies inconsistently with MEIS2 level when MEIS2 is overexpressed or downregulated. Our results suggest significant regulation, which supports the hypothesis that BACE1 expression may be affected by multiple factors. Notably, as a chronic progressive disease, the slow and sustained increase in MEIS2 levels should not be ignored in AD.

In addition to its role in regulating BACE1 expression, MEIS2 regulates the expression of AD‐related genes involving ABCA1, ERN1, IRS2, and NFKB1. The ABCA1 is a major cholesterol transporter highly expressed in the liver and brain. In the brain, ABCA1 is linked to Aβ clearance and contribute to maintaining vascular function through APOE (Christensen et al., 2004; Wang, Cui, et al., 2022). ERN1 (IRE1α) is a key factor in endoplasmic reticulum stress response and is related to Aβ generation and tau pathological changes in AD. The activation of ERN1 could induce endoplasmic reticulum stress, which might exacerbate neurodegeneration in AD (Cho et al., 2007; Lange‐Dohna et al., 2003; Rossner et al., 2006). IRS2 and NFKB1 affect AD through the insulin signaling pathway and neuroinflammation respectively (Nordestgaard et al., 2015; Saghafinia et al., 2021). Moreover, MEIS2 could also influence various AD‐related biological processes, including microRNA regulation, mitochondrial function, and oxidative phosphorylation, among others (Figure 6).

Taking into account the alterations of MEIS2 levels in the CSF and serum of AD patients, we analysed the diagnostic potential of MEIS2 for AD. Our findings indicate that MEIS2 exhibits potential diagnostic and differential diagnostic utility for AD (Figure S6).

In summary, MEIS2 is involved in AD pathogenesis by activating BACE1 transcription. MEIS2 inhibition ameliorates cognitive deficits and protects against Aβ deposition in APP/PS1 mice by repressing BACE1 expression. Our study provides a new pathological mechanism for AD and suggests that MEIS2 might be a promising early intervention target for AD treatment.

## AUTHOR CONTRIBUTIONS

YC, YW, and PW designed all experiments and analysed data. XZ and JL performed in vitro primary culture experiments. MC, JZ, YH, QS, and XW performed sample collection. YC and YW performed immunoblots, qRT‐PCR, ChIP, ELISA, IF, Luciferase assay, and behavioural experiments. CL performed cell culture. YC wrote the manuscript, and YW and PW edited the manuscript. All authors read and approved the final manuscript.

## FUNDING INFORMATION

Beijing Hospital Authority Youth Program (No. QML20230812), State Key Program of the National Natural Science Foundation of China (No. 82030064), National Natural Science Foundation of China (Nos. 81871714, 81901406, 82102487), Beijing Sail Plan for Talents Development (No. ZYLX202114), Beijing Key Clinical Specialty, HUIZHI Talent Leadership Development Program of Xuanwu Hospital (No. HZ2021PYLJ023).

## CONFLICT OF INTEREST STATEMENT

All other authors declare they have no competing interests.

## Supporting information


Data S1.

## Data Availability

RNA‐seq data has been uploaded to the GEO database.
