# Supplementary material for: Myeloid ectopic viral integration site 2 accelerates the progression of Alzheimer's disease
Source: Aging Cell. 2024 Jul 12;23(10):e14260. doi: 10.1111/acel.14260 (PMC11464116; doi:10.1111/acel.14260)
Supplement: Supplementary file 1 — Data S1. [file ACEL-23-e14260-s001.docx]

Supplementary Materials for

**Myeloid ectopic viral integration site 2 accelerates the progression of Alzheimer's disease**

Yuting Cui *et al.*

*Corresponding author. Email: wangyaqi0619@163.com; pcw1905@126.com

**This file includes:**

Figs. S1 to S6

Tables S1 to S8


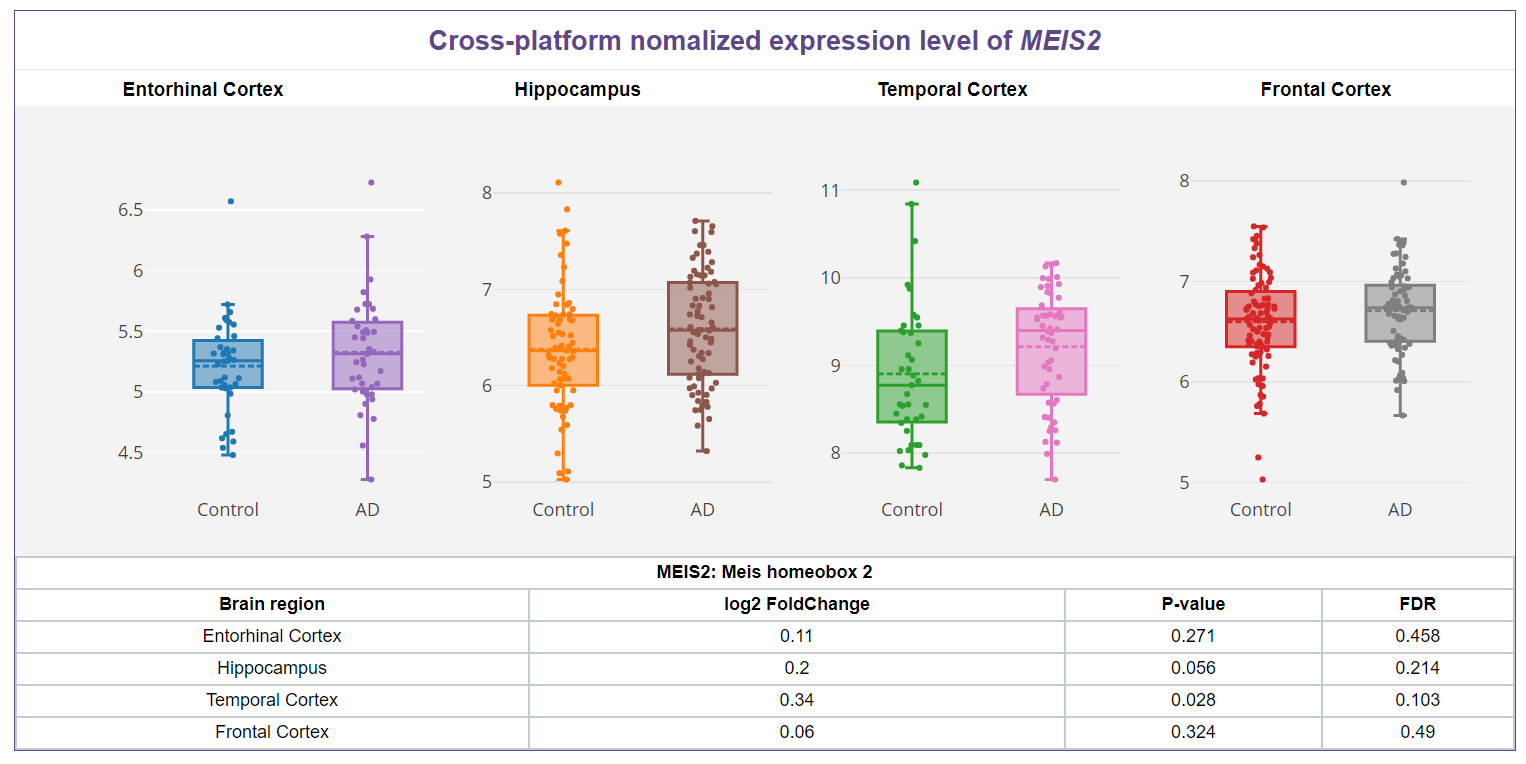


**Fig. S1. MEIS2 expression increased in Alzheimer's disease.** MEIS2 levels in the entorhinal cortex, hippocampus, temporal cortex and frontal cortex of patients with Alzheimer's disease were validated using AlzData.


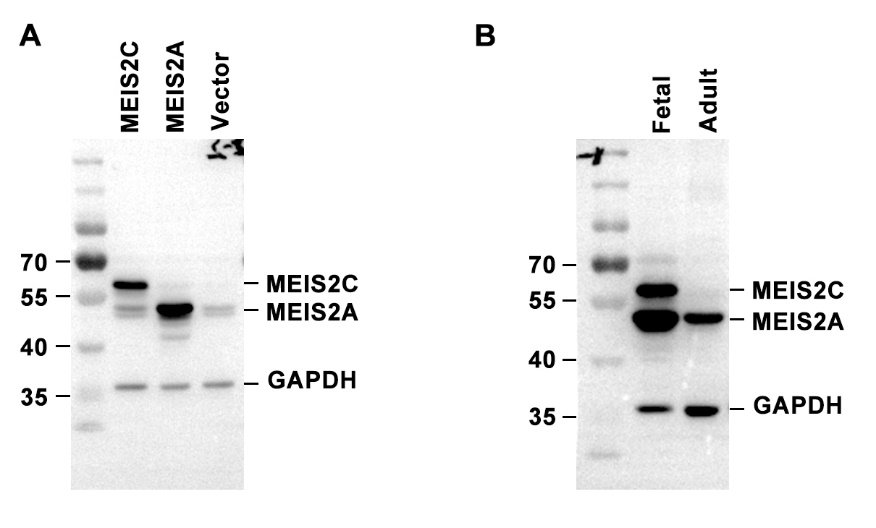


**Fig. S2. Transcript validation of MEIS2.**

(**A**) Validation of MEIS2 protein expression in N2a cells transfected with MEIS2A and MEIS2C plasmid.

(**B**) Validation of MEIS2 protein expression in the brain of fetal mouse and the 6-mouth adult mouse.


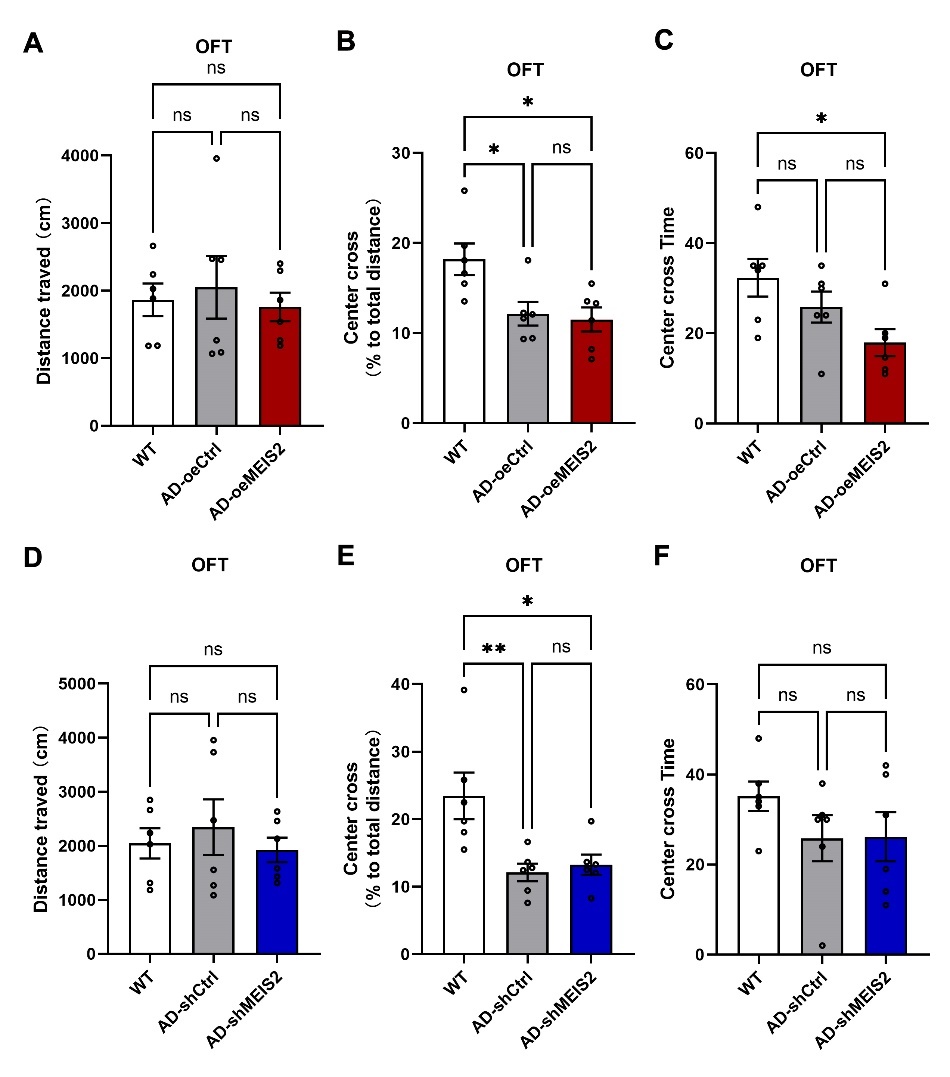


**Fig. S3. Mobility and anxiety were not affected by MEIS2 in the APP/PS1 mice. A-F** Open-field test results depict the total travelled distance (**A** and **D**), proportion of central distance (**B** and **E**) and the proportion of central time (**C** and **F**) of WT and APP/PS1 mice with different treatments. (**P* < 0.05, ***P* < 0.01). All data were presented as mean ± standard error of the mean (SEM) and were analysed by One-way ANOVA.


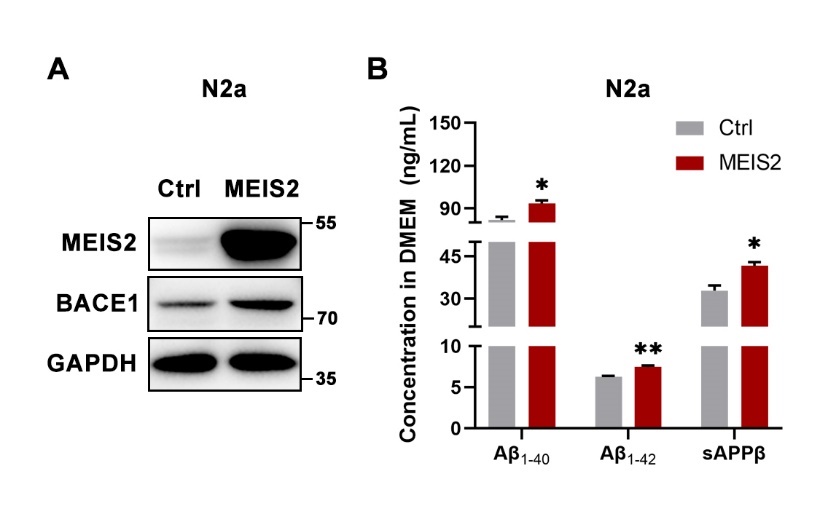


**Fig. S4. MEIS2 affects Aβ accumulation in N2a cells.** (**A**) Representative immunoblots of MEIS2 and BACE1 proteins in N2a cells transfected with MEIS2 plasmid and control vector. (**B**) The protein levels of Aβ1-40, Aβ1-42 and sAPPβ in the culture medium of the N2a cells transfected with MEIS2 plasmid and control vector (n = 3). (**P* < 0.05, ***P* < 0.01). Data are presented as mean ± SEM and analysed using Student’s *t-test*.


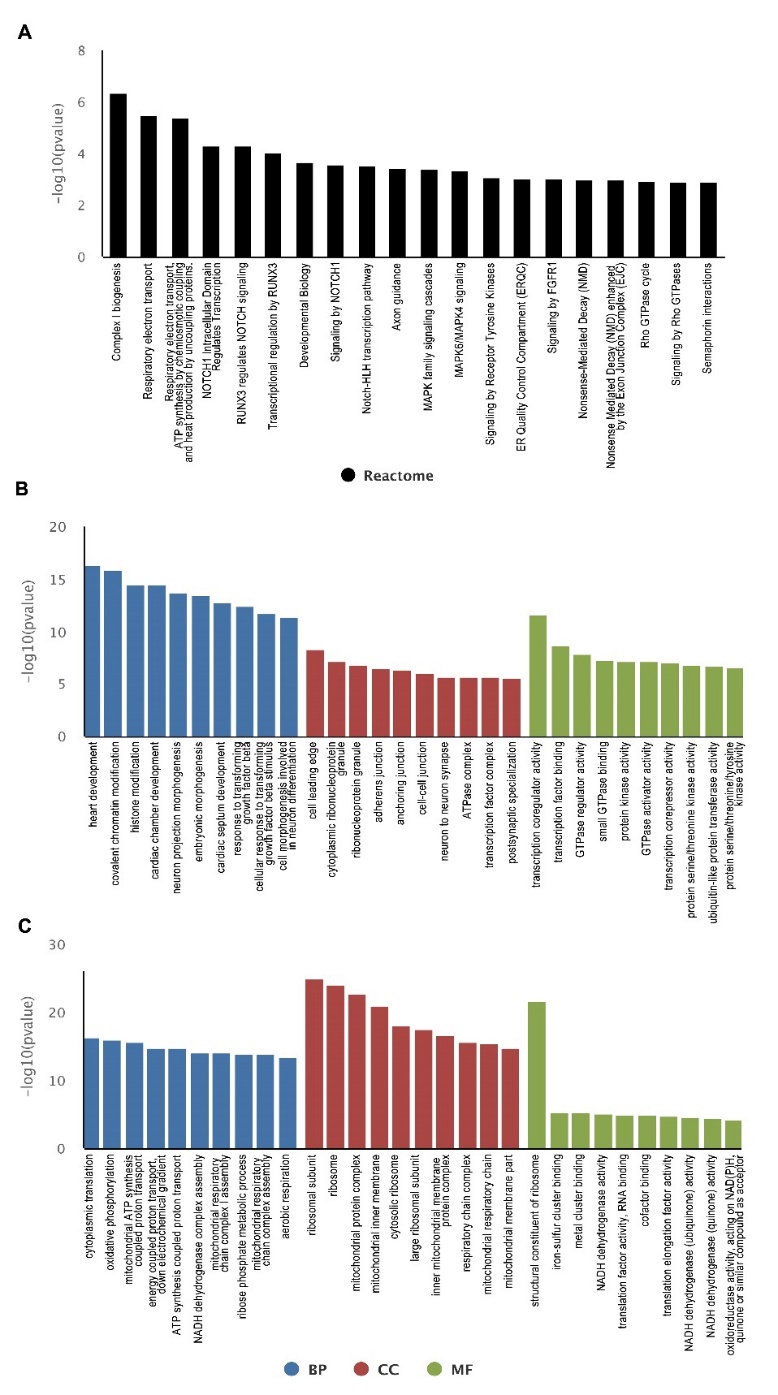


**Fig. S5. The Reactome pathway and GO analysis.** (**A**) Reactome pathway analysis of DEGs of MEIS2 overexpression. GO analysis for genes upregulated (**B**) and downregulated (**C**) in HT22 cells overexpressed MEIS2. Different colors indicate different GO classes. BP: biological process; CC: cellular component; MF: molecular function.


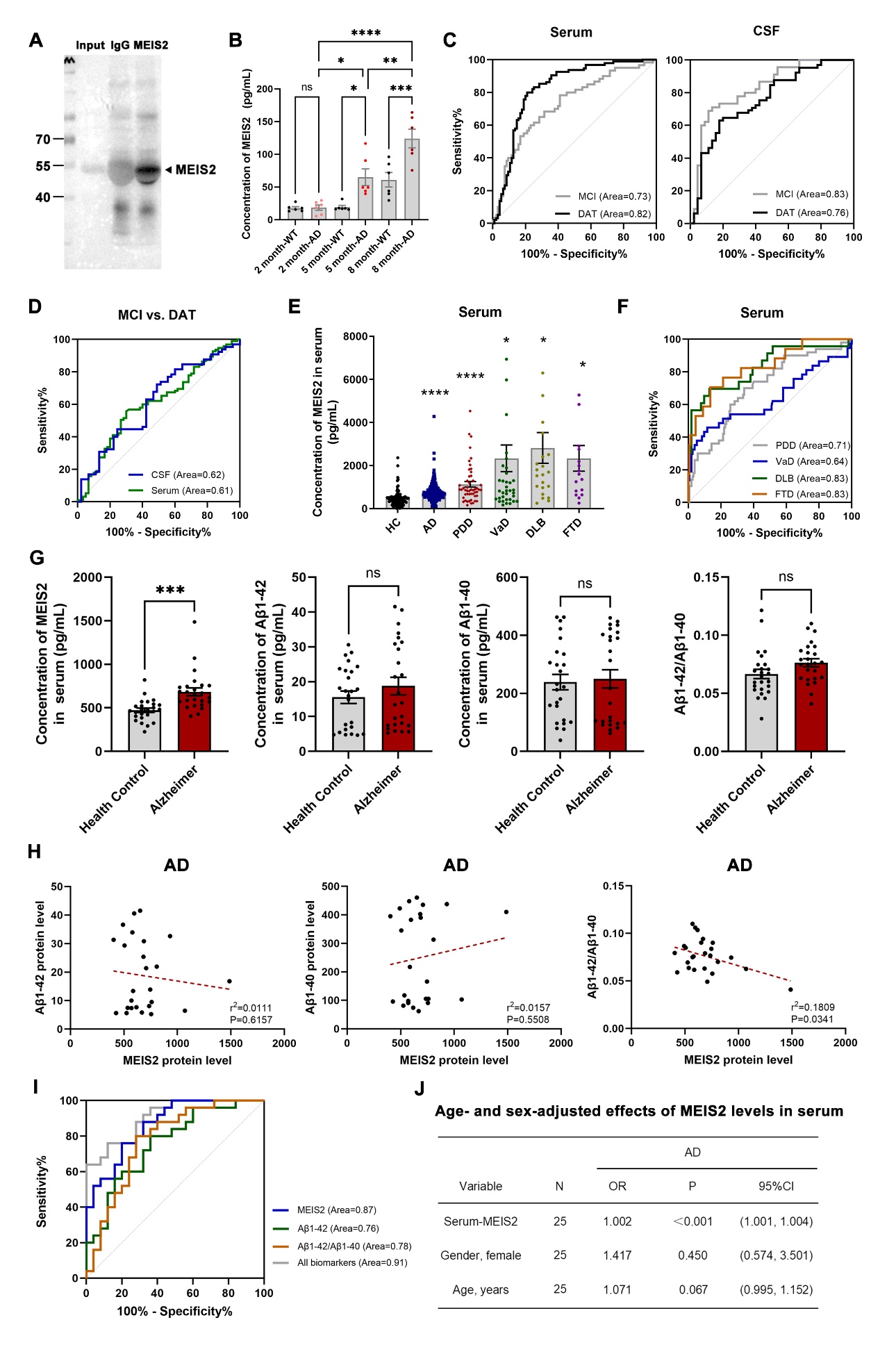


**Fig. S6. The potential value of MEIS2 as a diagnostic biomarker for AD.**

(**A**) Mixed CSF samples from different diseases were incubated with IgG or MEIS2 antibodies and beads overnight at 4 °C. Detected MEIS2 protein on beads by WB.

(**B**) MEIS2 levels in the serum of 2-month, 5-month, and 8-month APP/PS1 mice and WT mice (n = 6).

(**C**) The ROC curve of serum and CSF MEIS2 in the diagnosis of AD MCI-stage and DAT-stage from the normal cognition (NC) group.

(**D**) The ROC curve of serum and CSF MEIS2 in distinguishing AD MCI-stage from DAT-stage.

(**E**) MEIS2 levels in the sera of participants with AD (n = 155), Parkinson's disease dementia (PDD) (n = 50), vascular dementia (VaD) (n = 37), dementia with Lewy bodies (DLB) (n = 23), and frontotemporal dementia (FTD) (n = 17), and normal cognition (NC) (n = 95).

(**F**) The ROC curve of serum MEIS2 in the differential AD diagnosis from patients with PDD, VaD, DLB, and FTD, respectively. Data were presented as mean ± standard error of the mean (SEM).

(**G**) MEIS2, Aβ1-42, Aβ1-40 and Aβ1-42/Aβ1-40 levels in the sera of patients with Alzheimer's disease (n = 25) and participants with normal cognition (n = 25).

(**H**) Correlation between serum MEIS2 and Aβ1-42, Aβ1-40 and Aβ1-42/Aβ1-40 levels in patients with Alzheimer's disease.

(**I**) The ROC curve of serum MEIS2, Aβ1-42, Aβ1-42/Aβ1-40 and the combined index in the diagnosis of AD from the normal cognition (NC) group.

(**J**) Clinical data of age- and sex-adjusted effects of MEIS2 levels in serum.

Data are presented as mean ± SEM. (**P* < 0.05, ***P* < 0.01, ****P* < 0.001, *****P* < 0.0001). Data in **B** are analysed by One-way ANOVA; data in **E** are analysed by Brown-Forsythe ANOVA test; data in **G** is analysed by Student’s t-test; data in **H** is analysed by linear regression analysis. Clinical data are analysed by binary logistic regression.

Table S1. Clinical characteristics of AD and control cases.

| **Case ID** | **Age** | **Gender** | **Diagnosis** |
| --- | --- | --- | --- |
| Ctrl 1 | 72 | female | control |
| Ctrl 2 | 80 | male | control |
| Ctrl 3 | 76 | male | control |
| Ctrl 4 | 77 | male | control |
| AD 1 | 71 | male | AD |
| AD 2 | 86 | male | AD |
| AD 3 | 95 | female | AD |

Table S2. Characterization of patients in different CSF groups.

| \| **Characteristics** \| **Normal cognition (*n* = 45)** \| **MCI (*n* = 45)** \| **DAT (*n* = 65)** \| \| --- \| --- \| --- \| --- \| \| **Gender (n)** \| \| \| \| \| Male \| 23 \| 18 \| 27 \| \| Female \| 22 \| 27 \| 38 \| \| **Age** \|  \|  \|  \| \| Mean ± SEM \| 59.49 ± 1.12 \| 61.18 ± 0.90 \| 60.49 ± 0.65 \| \| **MMSE score** \|  \|  \|  \| \| Mean ± SEM \| - \| 26.20 ± 0.44 \| 15.75 ± 0.82**** \| \| **MoCA score** \|  \|  \|  \| \| Mean ± SEM \| - \| 21.64 ± 0.72 \| 11.48 ± 0.76**** \|   MCI: Mild cognitive impairment; DAT: Dementia of the Alzheimer type  *****P* < 0.0001, the data were analyzed by Student’s test, vs. MCI. |
| --- | --- | --- | --- | --- | --- | --- | --- | --- | --- | --- | --- | --- | --- | --- | --- | --- | --- | --- | --- | --- | --- | --- | --- | --- | --- | --- | --- | --- | --- | --- | --- | --- | --- | --- | --- | --- | --- | --- | --- | --- |

Table S3. Characterization of patients in different serum groups.

| **Characteristics** | **Health Control (*n* = 95)** | | **MCI**  **(*n* = 60)** | **DAT**  **(*n* = 95)** | **PDD**  **(*n* = 50)** | **VaD**  **(*n* = 37)** | **DLB**  **(*n* = 23)** | **FTD**  **(*n* = 17)** |
| --- | --- | --- | --- | --- | --- | --- | --- | --- |
| **Gender (n)** | |  |  |  |  |  |  |  |
| Male | | 44 | 31 | 36 | 32 | 20 | 13 | 8 |
| Female | | 51 | 29 | 59 | 18 | 17 | 10 | 9 |
| **Age** | |  |  |  |  |  |  |  |
| Mean ± SEM | | 65.96 ± 0.92 | 66.53 ± 1.25 | 66.45 ± 0.92 | 64.28 ±1.14 | 66.11 ± 1.60 | 70.30 ± 1.38 | 63.82 ± 1.63 |
| **MMSE score** | |  |  |  |  |  |  |  |
| Mean ± SEM | | - | 24.80 ± 0.51 | 16.28 ±0.66 **** | 26.66 ± 0.63 | 19.92 ± 0.99 | 19.48 ± 1.76 | 13.41 ± 2.03 |
| **MoCA score** | |  |  |  |  |  |  |  |
| Mean ± SEM | | - | 20.72 ± 0.63 | 11.33 ± 0.61**** | 21.40 ± 0.69 | 14.68 ± 0.99 | 14.35 ± 1.65 | 8.47 ± 1.71 |

MCI: Mild cognitive impairment; DAT: Dementia of the Alzheimer type

*****P* < 0.0001, the data were analyzed by Student’s test, vs. MCI.

Table S4. Target sequences of Short-hairpin RNA (shRNA) targeting MEIS2 (MEIS2-shRNA) and BACE1 (BACE1-shRNA).

| **Target sequences** |  |
| --- | --- |
| MEIS2-shRNA | 5’-CGGGACTGACAATTCTGCAAGTGAA-3 |
| BACE1-shRNA | 5’-CGTCATGATGGAAGGTTTCTA-3 |

Table S5. Primer sequences for real-time PCR and ChIP-qPCR.

| **Primers for real-time PCR** |  |
| --- | --- |
| **Gene** | **Primer Sequences** |
| mMEIS2 | F: 5’-AGAGACAAGGACGCAATC-3’ |
|  | R: 5’-GAAGGAGTCAGAGGAACAG-3’ |
| mBACE1 | F: 5’-ACATATCGAGACCTCCGAAAGG-3’  R: 5’-AACTTGTCCGATTCAGTGATGG-3 |
| mβ-actin | F: 5’-GGTCAGAAGGACTCCTATGTGG-3’  R: 5’-TGTCGTCCCAGTTGGTAACA-3’ |
| hMEIS2 | F: 5’-GAAAAGGTCCACGAACTGTGC-3’ |
|  | R: 5’-CTTTCATCAATGACGAGGTCGAT-3’ |
| hBACE1 | F: 5’-ACCAACCTTCGTTTGCCCAA-3’  R: 5’-TCTCCTAGCCAGAAACCATCAG-3’ |
| hGAPDH | F: 5’-GGTCGGAGTCAACGGATTTG-3’  R: 5’-GGAAGATGGTGATGGGATTTC-3’ |
| **Primers for ChIP-qPCR** |  |
| mBACE1 promoter Site 1 | F: 5’-TGAGGGCATTGGCAGAA-3’ |
|  | R: 5’-TGAGTATGTTGTGGAGTCTAC-3’ |
| mBACE1 promoter Site 2/ Site 3/ Site 4 | F: 5’-GAAGAAGCGTCTGTCTCT-3’ |
|  | R: 5’-ACATTCCAGCATACTCATTG-3’ |
| mBACE1 promoter Site 5 | F: 5’-ACACTGTGGCAAGAATCA-3’  R: 5’-CCTGAATCAGCATTGGTTAA-3 |

Table S6. Age- and sex-adjusted effects of MEIS2 levels in CSF.

|  |  |  |  | **MCI** |  |  |  |  | **DAT** |  |
| --- | --- | --- | --- | --- | --- | --- | --- | --- | --- | --- |
| **Variable** |  | **N** | **OR** | **P** | **95%CI** |  | **N** | **OR** | **P** | **95%CI** |
| **CSF-MEIS2** |  | 45 | 1.00 | ＜0.01 | (1.001, 1.004) |  | 65 | 1.00 | ＜0.01 | (1.001, 1.003) |
| **Gender, female** |  | 45 | 1.42 | 0.45 | (0.574, 3.501) |  | 65 | 1.35 | 0.47 | (0.598, 3.036) |
| **Age, years** |  | 45 | 1.07 | 0.07 | (0.995, 1.152) |  | 65 | 1.05 | 0.18 | (0.979, 1.117) |

Table S7. Age- and sex-adjusted effects of MEIS2 levels in serum.

|  |  |  |  | **AD** |  |  |  |  | **PDD** |  |  |  |  | **VaD** |  |
| --- | --- | --- | --- | --- | --- | --- | --- | --- | --- | --- | --- | --- | --- | --- | --- |
| **Variable** |  | **N** | **OR** | **P** | **95%CI** |  | **N** | **OR** | **P** | **95%CI** |  | **N** | **OR** | **P** | **95%CI** |
| **Serum-MEIS2** |  | 155 | 1.00 | ＜0.01 | (1.002, 1.004) |  | 50 | 1.00 | ＜0.01 | (1.002, 1.004) |  | 37 | 1.00 | ＜0.01 | (1.003, 1.005) |
| **Gender, female** |  | 155 | 1.06 | 0.83 | (0.616, 1.824) |  | 50 | 0.43 | 0.03 | (0.203, 0.914) |  | 37 | 0.64 | 0.29 | (0.274, 1.475) |
| **Age, years** |  | 155 | 0.98 | 0.45 | (0.958, 1.019) |  | 50 | 0.95 | 0.01 | (0.908, 0.989) |  | 37 | 0.96 | 0.11 | (0.914, 1.009) |

|  |  |  | **DLB** |  |  |  |  | **FTD** |  |
| --- | --- | --- | --- | --- | --- | --- | --- | --- | --- |
| **Variable** | **N** | **OR** | **P** | **95%CI** |  | **N** | **OR** | **P** | **95%CI** |
| **Serum-MEIS2** | 23 | 1.00 | ＜0.01 | (1.003, 1.005) |  | 17 | 1.00 | ＜0.01 | (1.003, 1.005) |
| **Gender,female** | 23 | 0.58 | 0.29 | (0.210, 1.590) |  | 17 | 0.81 | 0.71 | (0.270, 2.425) |
| **Age,years** | 23 | 1.02 | 0.55 | (0.959,1.082) |  | 17 | 0.93 | 0.03 | (0.874, 0.992) |

Table S8. Diagnostic ability of serum MEIS2 to discriminate between AD and non-AD diseases.

| **Groups** | **AUC (95% CI)** | **Cut-off Value (ng/ml)** | **p-value** | **Sensitivity (%)** | **Specificity (%)** |
| --- | --- | --- | --- | --- | --- |
| AD versus HC | 0.79 (0.72 to 0.85) | 543.60 | <0.01 | 75.48 | 77.89 |
| AD versus PD | 0.61 (0.52 to 0.71) | 820.00 | 0.02 | 60.00 | 67.10 |
| AD versus VaD | 0.60 (0.48 to 0.73) | 1198.00 | 0.06 | 43.24 | 89.03 |
| AD versus DLB | 0.79 (0.65 to 0.92) | 1510.00 | <0.01 | 60.87 | 96.13 |
| AD versus FTD | 0.77 (0.62 to 0.92) | 1221.00 | <0.01 | 64.71 | 89.03 |
